# Development of a set of novel binary expression vectors for plant gene function analysis and genetic transformation

**DOI:** 10.3389/fpls.2022.1104905

**Published:** 2023-01-12

**Authors:** Xiuyuan Wang, Chong Teng, Huitian Wei, Shuang Liu, Hongzhuan Xuan, Wentao Peng, Qianqian Li, Hongyan Hao, Qingya Lyu, Shanhua Lyu, Yinglun Fan

**Affiliations:** College of Agriculture, Liaocheng University, Liaocheng, China

**Keywords:** plant binary expression vector, *mScarlet-I* gene, visible selection marker, golden gate cloning, one-step digestion-ligation reaction

## Abstract

With the advent of multiple omics and Genome-Wide Association Studies (GWAS) technology, genome-scale functional analysis of candidate genes is to be conducted in diverse plant species. Construction of plant binary expression vectors is the prerequisite for gene function analysis. Therefore, it is of significance to develop a set of plant binary expression vectors with highly efficient, inexpensive, and convenient cloning method, and easy-to-use in screening of positive recombinant in *Escherichia coli*. In this study, we developed a set of plant binary expression vectors, termed pBTR vectors, based on Golden Gate cloning using *Bsa*I restriction site. Foreign DNA fragment of interest (FDI) can be cloned into the destination pBTR by one-step digestion–ligation reaction in a single tube, and even the FDI contains internal *Bsa*I site(s). Markedly, in one digestion–ligation reaction, multiple FDIs (exemplified by cloning four soybean *Glyma.02g025400*, *Glyma.05g201700*, *Glyma.06g165700*, and *Glyma.17g095000* genes) can be cloned into the pBTR vector to generate multiple corresponding expression constructs (each expression vector carrying an FDI). In addition, the pBTR vectors carry the visual marker, a brightness monomeric red fluorescent protein mScarlet-I, that can be observed with the unaided eye in screening of positive recombinants without the use of additional reagents/equipment. The reliability of the pBTR vectors was validated in plants by overexpression of *AtMyb75*/*PAP1* in tomato and *GUSPlus* in soybean roots *via Agrobacterium rhizogenes*-mediated transformation, promoter activity analysis of *AtGCSpro* in *Arabidopsis via A. tumefaciens*-mediated transformation, and protein subcellular localization of the *Vitis vinifera* VvCEB1_opt_ in tobacco, respectively. These results demonstrated that the pBTR vectors can be used in analysis of gene (over)expression, promoter activity, and protein subcellular localization. These vectors will contribute to speeding up gene function analysis and the process of plant molecular breeding.

## Introduction

With the rapid advance of DNA sequencing technology, genomes of 800 terrestrial plants have been sequenced (Marks et al., 2021; Lu et al., 2021; Kamal et al., 2022; Peng et al., 2022; Sun et al., 2022). Concomitantly, the rapidly developing of multiple omics and Genome-Wide Association Studies (GWAS) in diverse plant species, genome-scale functional analyses of large numbers of candidate genes will have been conducted. Construction of plant binary expression vector is the prerequisite for gene function analysis. Therefore, it is important to develop a set of cost-efficient, easy-to-use, and highly efficient plant binary expression vectors. In previous studies, a series of binary expression vectors have been developed, such as pBIN19 and its derivatives (the binary pBI series) (Barton et al., 1983; Bevan, 1984; Jefferson et al., 1987), pGreen/pSoup and its derivative (pCLEAN), pPZP and its derivatives (pCAMBIA series) (Hajdukiewicz et al., 1994; van Engelen et al., 1995; Hellens et al., 2000; Zheng et al., 2001; Thole et al., 2007; Belknap et al., 2008), and Gateway binary vectors (e.g. pGWBs, pMDCs, pEarleyGate) (Curtis and Grossniklaus, 2003; Nakagawa et al., 2007; Karimi et al., 2007). In these expression vectors, to achieve the assembly of the foreign DNA fragment of interest (FDI) into the plant binary expression vectors, the main molecular cloning approaches available primarily include homologous recombination and the traditional restriction digestion–ligation methods. Homologous recombination technology that includes *in vivo* and *in vitro* assemblies depends on short homologous ends. The *in vivo* assembly method (Mosberg et al., 2010; Fu et al., 2012) is simple in operation and looks attractive, but it does not become popular and be widely used (Watson and García-Nafría, 2019). *In vitro* assembly strategies include a variety of homology-based methods, such as ligation-independent cloning (LIC) (Aslanidis and de Jong, 1990), Gateway cloning (Hartley et al., 2000; Walhout et al., 2000), SLIC (Li and Elledge, 2007), SliCE (Zhang et al., 2012; Motohashi, 2015), In-fusion cloning (Benoit et al., 2006; Sleight et al., 2010), Gibson assembly (Zhu et al., 2007; Gibson et al., 2009), and Nimble cloning (Yan et al., 2020). Homology-based cloning relies on small homologous sequences at the termini of DNA fragments generally introduced by PCR to drive the assembly of desired circular recombinants. It is simple, sequence-independent, and directional. However, it is costly to assemble genome-scale candidate FDIs into expression vectors. Compared to *in vivo* and *in vitro* homology-based cloning methods, the traditional restriction digestion–ligation method is low-cost, simple, popular, and widely used. However, it is sequence-dependent, and dozens of diverse restriction enzymes need to be stored in a lab to be able to construct different vectors. Although directional cloning can be achieved by combining two different restriction endonucleases, isocaudomers should be carefully considered to evade DNA molecular self-ligation, and some restriction enzymes show buffer incompatibility. Furthermore, digestion by restriction enzymes and ligation *via* ligase reactions are separately completed, and an additional DNA purification step is also required. Therefore, it is time-consuming, inefficient, and sequence-dependent. Additionally, the DNA purification step (requiring the purchase of a kit) can result in a partial loss of FDI and vector.

Engler et al. (2008) reported a Golden Gate cloning method that overcomes some shortcomings of this traditional restriction digestion–ligation method. It uses only a single restriction endonuclease, and entails a one-step digestion–ligation reaction without a DNA purification step. Based on Golden Gate cloning, Lampropoulos et al. (2013) and Emami et al. (2013) developed several plant expression vectors that can rapidly and sequentially integrate multiple pre-made modules (including promoter, FDI, terminator, selection marker, or reporter gene, etc.) into a plant expression vector. However, the expression vectors can not be directly used for cloning of FDI (requiring multiple expression-related elements integrated and arranged in a designed order). It is greatly complex in operation to clone FDI carrying one or several internal restriction enzyme site(s) into the expression vector (Engler et al., 2008; Emami et al., 2013). Furthermore, the screening of positive colonies in *E. coli* depends on the additional chemicals reagents. In this study, we developed a series of novel, high-efficiency plant binary expression vectors, termed pBTR vectors (representing initials of *Bsa*I, T4 ligase, Reddish-pink colony of *E*. *coli*), based on Golden Gate cloning (Engler et al., 2008) for *Agrobacterium*-mediated plant transformation. Only a single *Bsa*I restriction enzyme is used, and the FDI can be directly and conveniently cloned into the destination pBTR expression vector with almost sequence-independence and no additional DNA purification step by a one-step digestion–ligation reaction. The associated costs are substantially low. Significantly, in a single digestion–ligation reaction, multiple FDIs can be independently cloned into pBTR vector to generate multiple corresponding expression vectors (each expression vector carrying an FDI). In addition, the pBTRs allow for directional cloning, and carry directly observable visual selection marker that can be used in screening positive transformants in *E. coli* according to the colony color by the unaided eye. These vectors will pave the way for accelerating the speed of gene function studies and the process of plant molecular breeding.

## Results

### Development of the pBTRs

**Figure 1:**
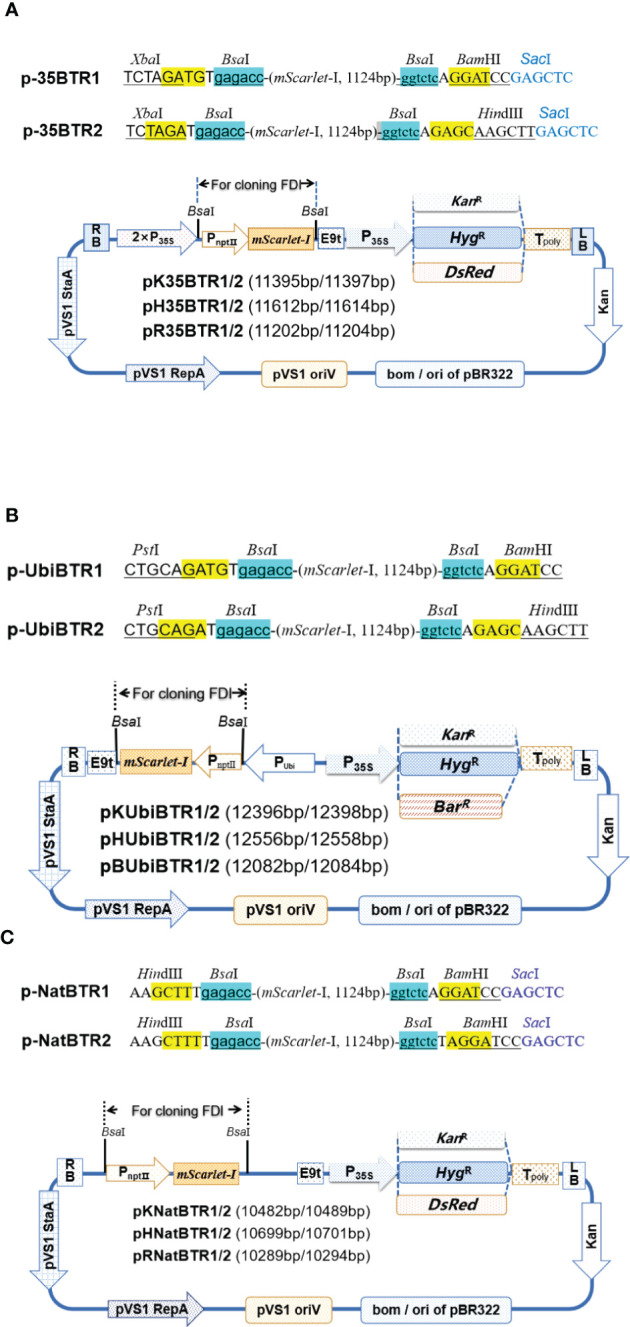
Diagrams of (over)expression vectors. Gene (over) expression vectors driven by *CaMV 35S* based on the pHSE401/pKSE401 backbone **(A)**. *Ubiquitin* promoter based on the pYLCRISPR/Cas9Pubi-series of vectors backbone **(B)**. Native promoter based on the pHSE401/pKSE401 backbone **(C)**. Kan^R^, *kanamycin* resistance gene; Hyg^R^, *hygromycin* resistance gene; Bar^R^, *basta* resistance gene. The p*NptⅡ::*m*Scarlet*-I expression cassette was used for a visual selection marker in *E coli*. The two *Bsa*I recognition sites and neighbor sequences designed for cloning FDI are shown at the top of the vectors. The key restriction sites for further screening of positive recombinants are given. The *Kan*^R^, *Hyg*^R^, *DsRed*, and *Bar*^R^ genes driven by *CaMV 35S* were used as selection markers/reporters in plant. 5’ overhangs (sticky end) produced after digestion by *Bsa*I are highlighted in yellow.

**Figure 2:**
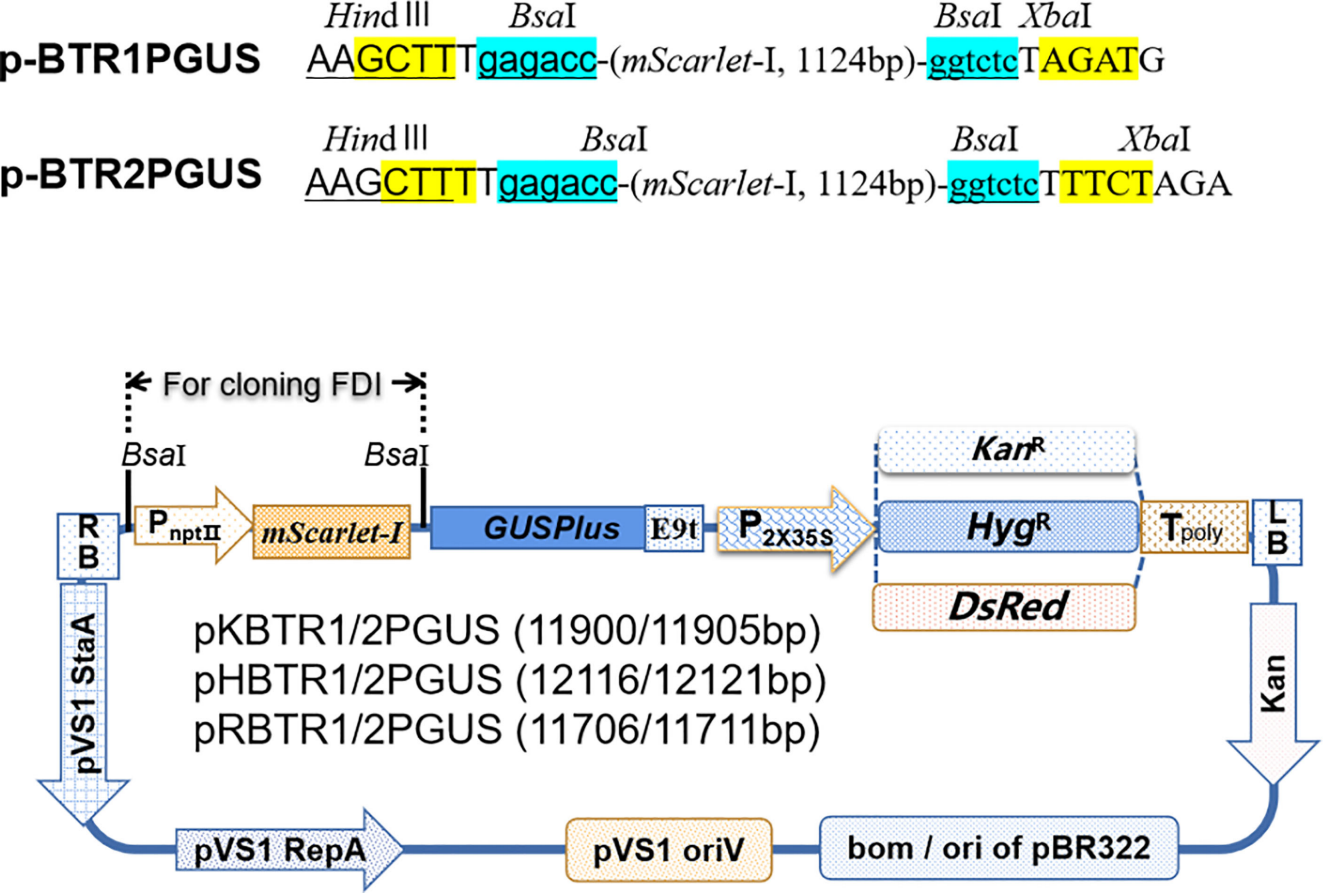
Diagrams of promoter analysis vectors. *Hin*dIII and *Xba*I restriction enzyme recognition sites for further screening of positive recombinants in *E*. *coli* are given. The vectors pKBTR1/2PGUS, pHBTR1/2PGUS, and pRBTR1/2PGUS carrying a *Kan*^R^, *Hyg*^R^, and *DsRed* gene, respectively, driven by *CaMV 35S* (enhancer were deleted) were used as selection markers in plant. Sticky ends produced after digestion by *Bsa*I are highlighted in yellow.

**Figure 3:**
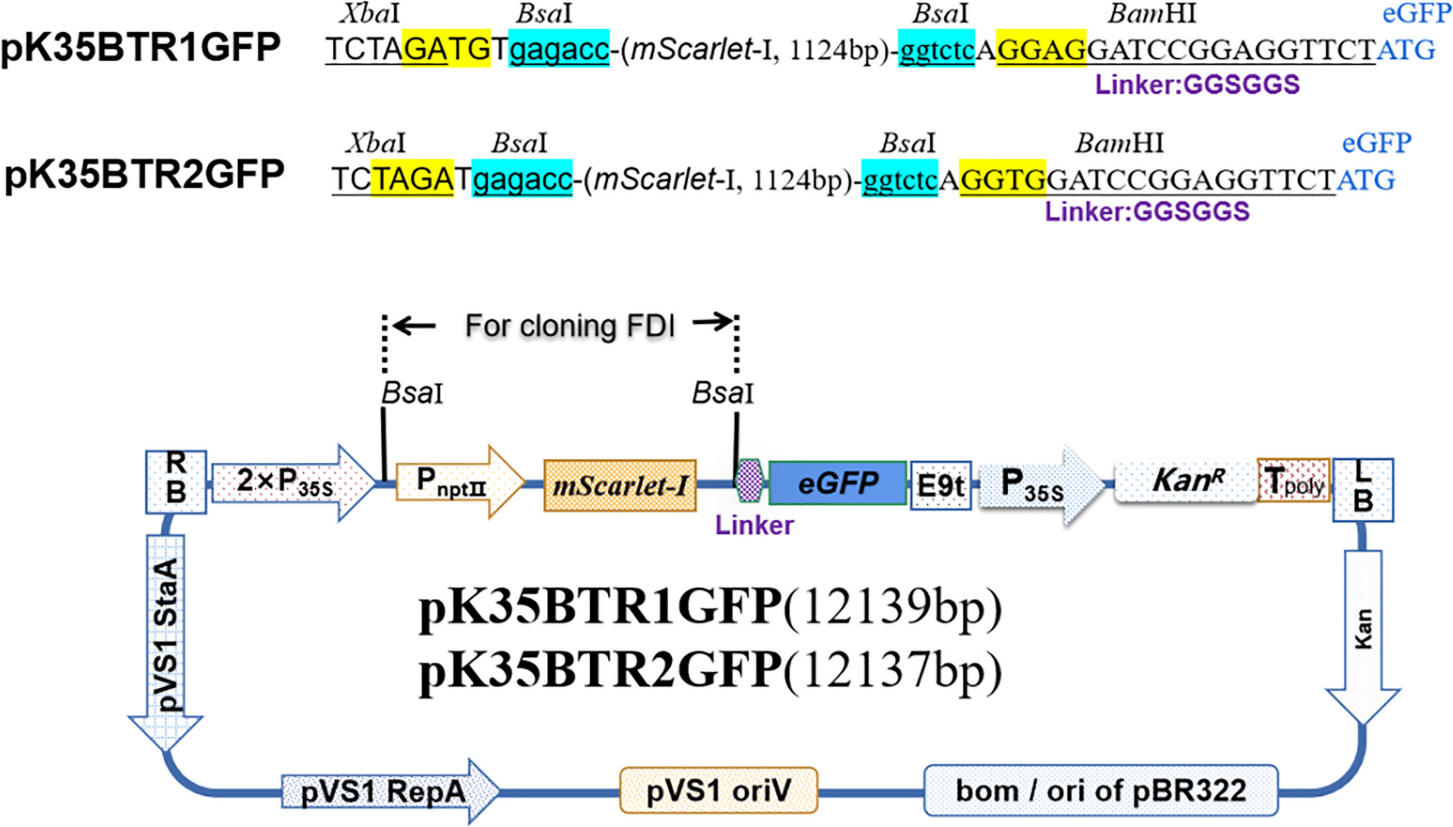
Diagram of subcellular localization vector.

To achieve simple one-step cloning based on the digestion–ligation method, we developed a series of novel vectors including vectors targeted at (over)expression of genes (**Figure 1**), promoter analysis (**Figure 2**), and subcellular localization (**Figure 3**), based on the Golden Gate cloning method with only one restriction endonuclease *Bsa*I (the cheapest type IIs restriction endonuclease) and T4 ligase required. For each vector type, we developed two sets, termed pBTR1 and pBTR2, respectively. pHSE401/pKSE401 (Xing et al., 2014) and pYLCRISPR/Cas9Pubi-B/H/N (Ma et al., 2014) which derived from pCAMBIA-based binary expression vectors, were used as backbones with *Bsa*I recognition sequences eliminated. These have been widely used well in both *Agrobacterium tumefaciens*- and *Agrobacterium rhizogenesis*-mediated plant transformation. *Bsa*I recognizes the 5’-GGTCTC(N1)/(N5)-3’ site and cuts outside of its recognition sequence at the 3’-end of DNA molecular, and thereby, producing a 5’ overhang 4 nt in length. We incorporated two tandem *Bsa*I recognition sites arranged in reverse orientation with their neighboring sequences into the pBTR vectors. After a given vector is digested by *Bsa*I, it lacks the original *Bsa*I recognition site and has different 5’ non-palindromic overhangs at each terminal. For example, in the (over)expression of gene vectors, the p-35BTR1 set has CATC (5’-3’) and GGAT (5’-3’) 5’ overhangs while p-35pBTR2 has TCTA (5’-3’) and GAGC (5’-3’) 5’ overhangs (**Figure 1A**). Therefore, pBTR1 and pBTR2 cannot be self-religated after the single *Bsa*I digestion (**Figures 1**–**3**). This prevents the generation of false-positive colonies of *E. coli* (empty vector) and ensures that FDI is integrated into the destination vector in a designed orientation.

**Figure 4:**
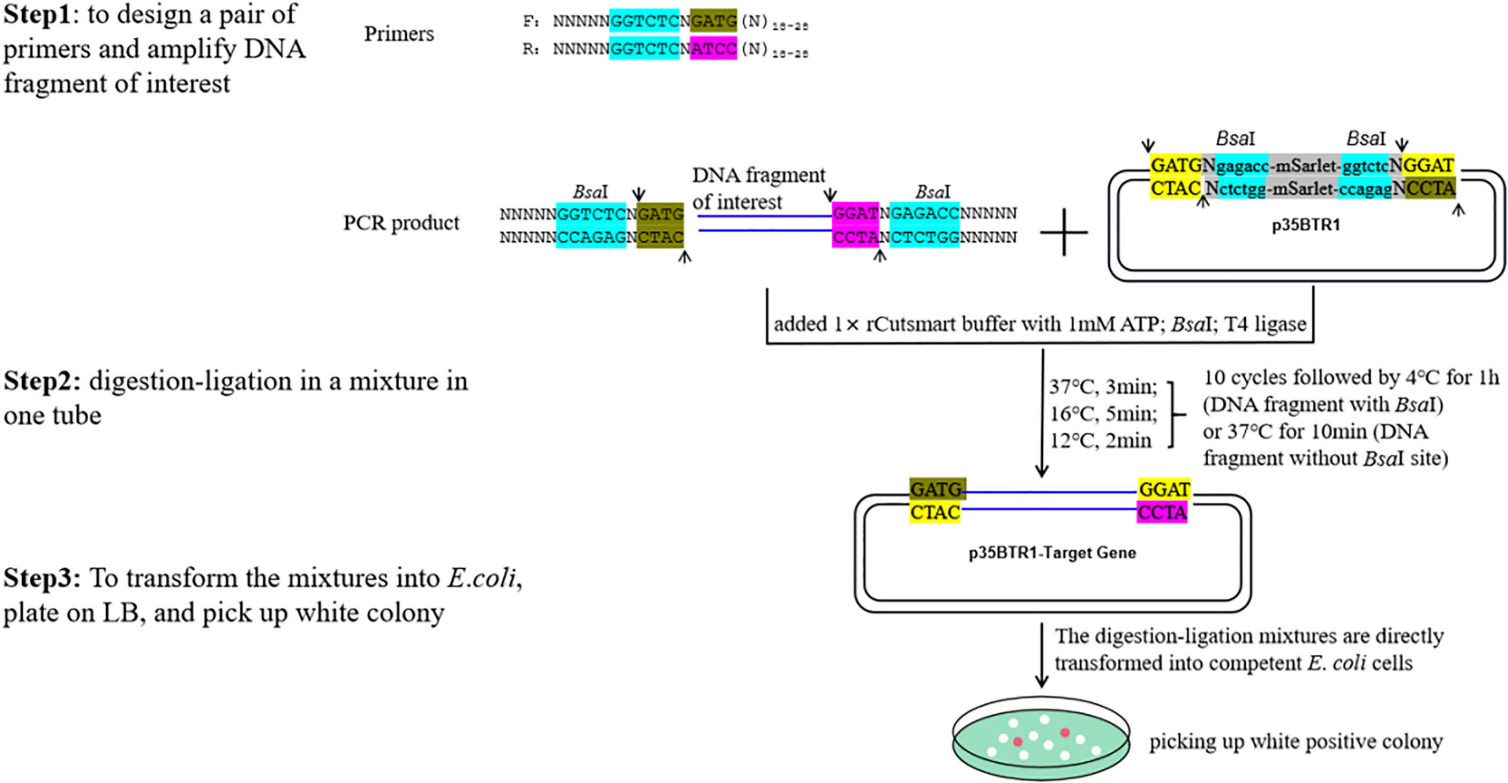
An example of the schematic of the construction of a pBTR vector using one-step digestion–ligation. N_18-28_ represents any 18–28 nt sequences (forward or reverse primers) paired with the FDI. Arrows indicate the digestion site by *Bsa*I. The *Bsa*I recognition sites are shown in blue. Two tandem *Bsa*I recognition sites and neighboring sequences were incorporated into the pBTR vectors. FDI was cloned into p35BTR1, generating a recombinant product lacking the original *Bsa*I restriction site.

Next, FDI was added with adapters carrying the *Bsa*I recognition sequences at the forward and reverse primer 5’ ends introduced by PCR for cloning into the pBTR1 or pBTR2 vector. This was done to generate specific overhangs at each end of each FDI that complement those of the corresponding destination vectors in the desired orientation after digestion by *Bsa*I. The adapter sequences applied for different expression vectors are shown in **Table 1**. The FDI and destination vector were concatenated together using T4 DNA ligase. The destination vector and the PCR product were digested and then ligated in a reaction mixture in one tube (**Figure 4**). For FDI carrying one or more internal *Bsa*I site(s), the digestion–ligation step was performed in a thermocycler with the following program: 10 cycles at 37°C for 3 min, at 16°C for 3 min, and at 12°C for 2 min, followed by a final incubation step at 4°C for 1 h (holding ligation reaction step). For FDIs carrying no internal *Bsa*I recognition sequences, the same program was employed, but the final incubation step was at 37°C for 10 min (holding the restriction enzyme digestion reaction step) to decrease the number of empty vectors. The resulted digestion–ligation mixtures were directly transformed into competent *E*. *coli* cells to obtain the desired clones (**Figure 4**).

**Table 1:** Adapter sequences should be added in the primer 5’ end.

pBTRs plant binary expression vectors	adapter sequences in the forward primer 5’ end (5’-3’)	adapter sequences in the reverse primer 5’ end (5’-3’)
pH35BTR1/pK35BTR1/pR35BTR1 pBUbiBTR1/pHUbiBTR1/pKUbiBTR1	NNNNGGTCTCNGATG	NNNNGGTCTCNATCC
pH35BTR2/pK35BTR2/pR35BTR2	NNNNGGTCTCNTAGA	NNNNGGTCTCNGCTC
pBUbiBTR2/pHUbiBTR2/pKUbiBTR2	NNNNGGTCTCNCAGA	NNNNGGTCTCNGCTC
pHNatBTR1/pKNatBTR1/pRNatBTR1	NNNNGGTCTCNGCTT	NNNNGGTCTCNATCC
pHNatBTR2/pKNatBTR2/pRNatBTR2	NNNNGGTCTCNCTTT	NNNNGGTCTCNTCCT
pHBTR1PGUS/pKBTR1PGUS/pRBTR1PGUS	NNNNGGTCTCNGCTT	NNNNGGTCTCNATCT
pHBTR2PGUS/pKBTR2PGUS/pRBTR2PGUS	NNNNGGTCTCNCTTT	NNNNGGTCTCNAGAA
pK35BTR1GFP	NNNNGGTCTCNGATG	NNNNGGTCTCNCTCC
pK35BTR2GFP	NNNNGGTCTCNTAGA	NNNNGGTCTCNCACC

**Figure 5:**
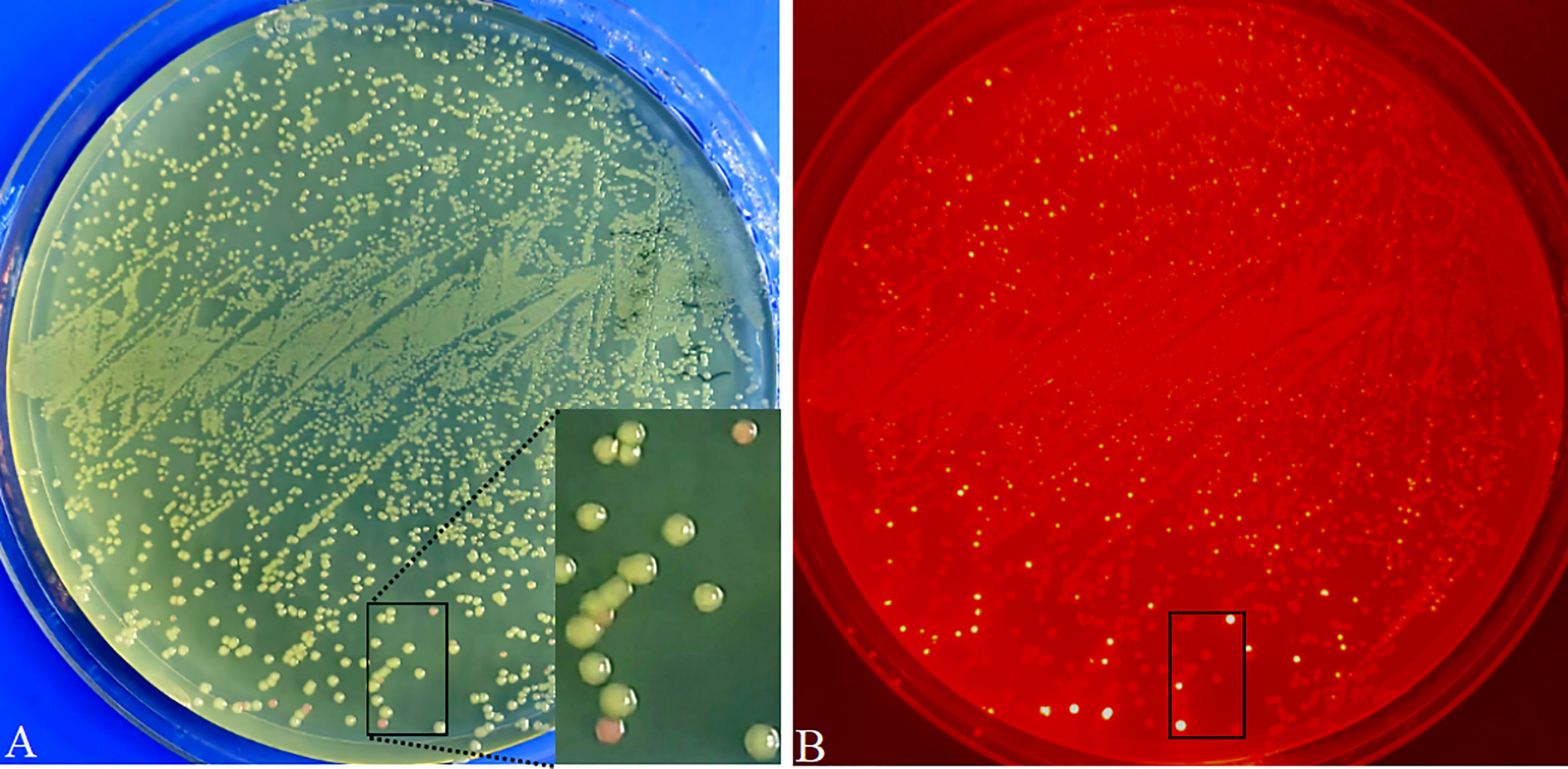
Visual screening of positive recombinants and cloning efficiency analysis in *E coli* transformed with pR35BTR2-*GmbHLH293*. Directly visual screening of recombinants by the naked eye under natural light. Positive recombinants are white color, while non-recombinants are reddish-pink **(A)**. *E coli* transformant colonies were observed under green excitation; non-recombinants are bright red **(B)**.

In the pBTR1/2, a visual selection marker, the *mScarlet-I* gene (Bindels et al., 2017) driven by the *neomycin phosphotransferase* gene promoter (P*_nptⅡ_*) was inserted between two *Bsa*I sites. The expression of *mScarlet-I* in *E*. *coli* results in a reddish-pink colony formed from negative transformants, while FDI replaced the expression cassette of P*_nptⅡ_*_::_ *mScarlet-I* from positive transformants and the positive colonies were white color (**Figures 1**–**5**). The purpose of this marker designed was to distinguish non-recombinants (reddish-pink colony) from positive recombinants (white colony) with the unaided eye without using any other reagent, substrate, or equipment. **Figure 4** presents a schematic depicting vector construction (p35BTR1 used as an example).

### Analyses of cloning efficiency in *E*. *coli* and validation of the gene (over)expression vectors in *planta*

*CaMV 35S* and *ubiquitin* promoters are highly active in most transgenic plant cells and are the most widely used to drive gene overexpression in dicots and monocots. Therefore, based on pHSE401/pKSE401 and pYLCRISPR/Cas9Pubi-B/H/N backbones, we created two series of plant binary overexpression vectors, termed p-35BTR1/2 (**Figure 1A**) and p-UbiBTR1/2 (**Figure 1B**), respectively, where *CaMV 35S* and *ubiquitin* promoters were used to drive candidate gene expression. To facilitate the screening of transgenic-positive plants, we developed 12 different overexpression vectors including six the p-35BTR1/2 series and six p-UbiBTR1/2 series, respectively, with different selection markers or reporter genes. The p-35BTR1/2 series included pK35BTR1/2 (carrying the *kanamycin* resistance selection marker gene, *Kan*^R^), pH35BTR1/2 (carrying the *hygromycin* resistance selection marker gene, *Hyg*^R^), and pR35BTR1/2 (carrying the *DsRed* fluorescent protein reporter gene, *DsRed*), respectively (**Figure 1A**). The p-UbiBTR1/2 series included pKUbiBTR1/2 (*Kan*^R^), pHUbiBTR1/2 (*Hyg*^R^), and pBUbiBTR1/2 (carrying the *basta* resistance gene, *Bar*^R^) (**Figure 1B**). Furthermore, we also created a series of plant binary expression vectors driven by a native promoter based on pHSE401/pKSE401 backbones (Xing et al., 2014), termed p-NatBTR1/2. This included p-NatBTR1/2 six vectors, pKNatBTR1/2 (*Kan*^R^), pHNatBTR1/2 (*Hyg*^R^), and pRNatBTR1/2 (*DsRed*) (**Figure 1C**).

To verify the availability and cloning efficiency of these vectors, *Arabidopsis AtMyb75*/*PAP1* (GenBank No. AY519563; no internal *Bsa*I recognition site) (Fan et al., 2020a), *GUSPlus* (cloned from pCAMBIA1305.1; carrying two internal *Bsa*I recognition sites), and soybean *GmbHLH293* (*Glyma.01g068600*; carrying two internal *Bsa*I sites and one *Bsa*I site producing a palindromic sticky end) were cloned into pK35BTR1, pK35BTR2, and pR35BTR2 vectors, respectively, followed by the protocol described in **Figure 4** and also described in detail in the “Material and methods” section. The digestion–ligation mixtures were directly transformed into competent *E*. *coli* cells. There were ~10, 000 colonies obtained with 100 µL *E.coli* chemically competent cells transformed with pK35BTR1*-AtMyb75* or pK35BTR2*-GUSPlus* using 20 ng vector plasmid. The number of pR35BTR2-*GmbHLH293* colonies was ~2, 000, less than the pK35BTR1*-AtMyb75* and pK35BTR2*-GUSPlus* transformants, possibly because *GmbHLH293* gene carrying one internal *Bsa*I site produced a palindromic sticky end. The desired recombinants appeared as white colonies, whereas non-recombinants were reddish-pink (**Figure 5**). These different colonies were easily and conveniently distinguished from each other by the naked eye under natural light (**Figure 5A**). The red fluorescence from these reddish-pink colonies can also be observed under the control of green excitation light due to the expression of *mScarlet-I* (**Figure 5B**). We randomly selected 8 white colonies to further confirm that they were positive recombinants *via* restriction enzyme digestion (**Figure S1A**) and Sanger sequencing (**Figure S1B**). The results indicated that these colonies were correctly identified using our new method. All such colonies showed 100% transformants with FDI inserted (**Figures S1A, B**). These results also show that *mScarlet-I* can serve as a reliable marker for highly efficient selection of positive colonies based on colony color. More than 90% of colonies transformed with the pK35BTR1*-AtMyb75* (103 white colonies/103 total colonies, 100%, **Figure S1C**), pK35BTR2*-GUSPlus* (124/124 total colonies, 100%, **Figure S1D**), and pR35BTR2-*GmbHLH293* (236 white colonies /262 total colonies, 90%, **Figure 5**) recombinant vectors were positive colonies.

**Figure 6:**
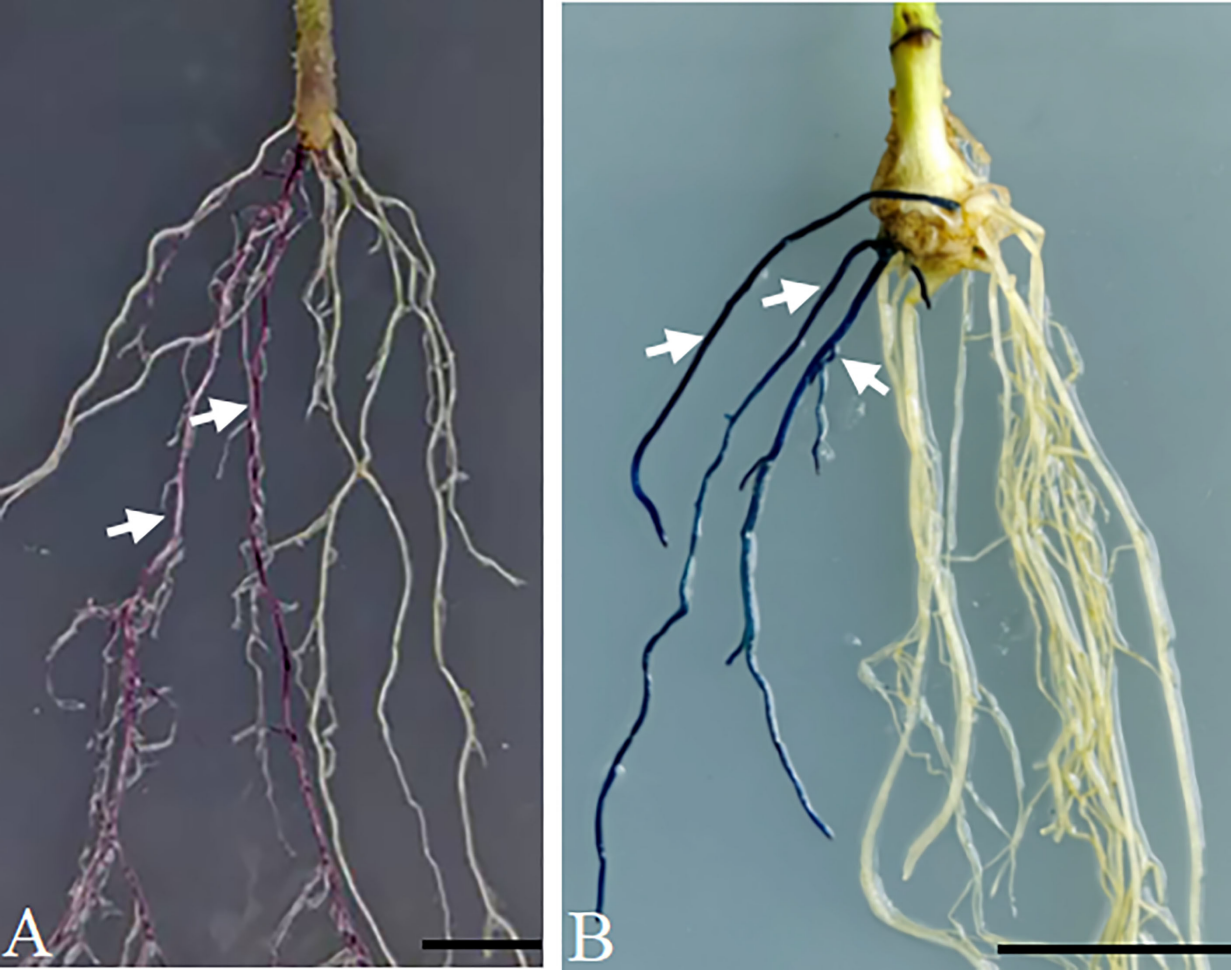
Verification of the overexpression vector p-35BTR1/2 in plants. Anthocyanin accumulated in the transgenic hairy roots of tomato transformed with pK35BTR1*-AtMyb75* **(A)**. The GUS signal was observed in the transgenic hairy roots of soybean transformed with pK35BTR2*-GUSPlus* **(B)**. Arrows indicate the transgenic roots; bars=1 cm.

To validate in *planta* expression of the cloned gene in the pBTR1/2 vector, the pK35BTR1*-AtMyb75* and pK35BTR2*-GUSPlus* plasmids were transformed into *A. rhizogenes strain K599*, and positive K599 carrying the corresponding plasmid were used to infect the hypocotyl of tomato and soybean, respectively, and to induce the generation of transgenic roots using one-step *A*. *rhizogenes-*mediated transformation (Fan et al., 2020a). Purple/red-colored anthocyanin accumulated in 35S::*AtMyb75* transgenic tomato roots but not in non-transgenic roots (**Figures 6A**, **S2A**). This is consistent with previous reports that overexpression of *AtMyb75*/*PAP1* in some plant roots can induce the accumulation of purple/red-colored anthocyanin in roots (Zuluaga et al., 2008; Fan et al., 2020b). As expected, the GUS signal was observed in overexpression-*GUSPlus* hairy roots of soybean (**Figures 6B**, **S2B**). These results indicate that pBTR1/2 overexpression vectors are reliable plant binary vectors for *Agrobacterium*-mediated plant transformation.

### Validation of promoter analysis vectors in *planta*

**Figure 7:**
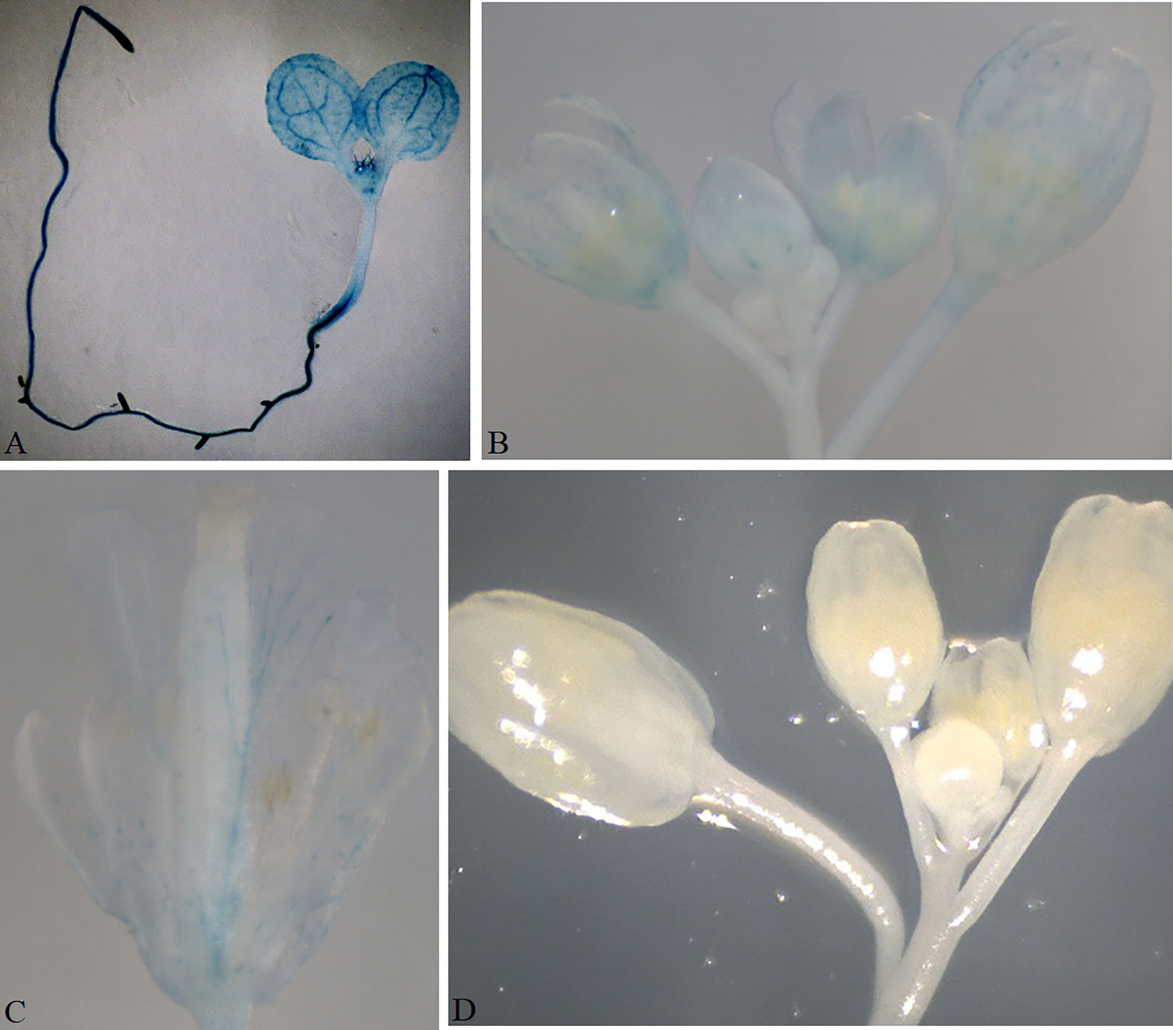
Validation of the promoter analysis vector in *Arabidopsis*. The GUS signal was observed in the cotyledon, shoot apical meristem, and the whole root of a 10-day-old transgenic seedling transformed with pRBTR1PGUS-*AtGCS* **(A)**. GUS expression in an inflorescence **(B)**; GUS expression in a single flower **(C)**. No GUS signal was observed in the *Arabidopsis* transformed with an empty pRBTR1PGUS vector as a negative control **(D)**.

Plant growth and development involve the temporal and spatial expression of specific gene subsets in response to various physiological and environmental factors mediated by complex signal transduction pathways. Promoter functional analyses can provide evidence of the functional action of genes by appraising the spatial and temporal domains of gene expression. The *GUSPlus* gene is often used as a reporter gene in such analyses. Here, we developed six promoter analysis vectors, including pKBTR1/2PGUS (*Kan*^R^), pHBTR1/2PGUS (*Hyg*^R^), and pRBTR1/2PGUS (*DsRed*) (**Figure 2**). In these vectors, the two internal *Bsa*I recognition sites carried in the *GUSPlus* gene were eliminated. To analyze promoter function and validate these vectors, an *Arabidopsis thaliana* gamma-*glutamylcysteine synthetase* gene (termed *AtGCS*; GenBank no. AF068299.1) promoter showing highly active in broad eudicots roots (Liu et al., 2022) but not characterized in *A. thaliana*, was cloned into the pRBTR1PGUS vector and transformed into wild-type *A. thaliana* by *A. tumefaciens*-mediated transformation using flower dip method (Clough and Bent, 1998). The *AtGCS* promoter can drive *GUSPlus* expression of transgenes in the cotyledon, shoot apical meristem, inflorescence, and vascular areas of sepal and petal tissues, besides roots (**Figures 7**, **S2C**). This expression pattern is consistent with that of the TAIR (The Arabidopsis Information Resource, http://www.arabidopsis.org) database. The results indicated that the pBTR1/2 vector sets for promoter analysis work well in plants *via Agrobacterium*-mediated plant transformation.

### Verification of protein subcellular localization vectors

**Figure 8:**
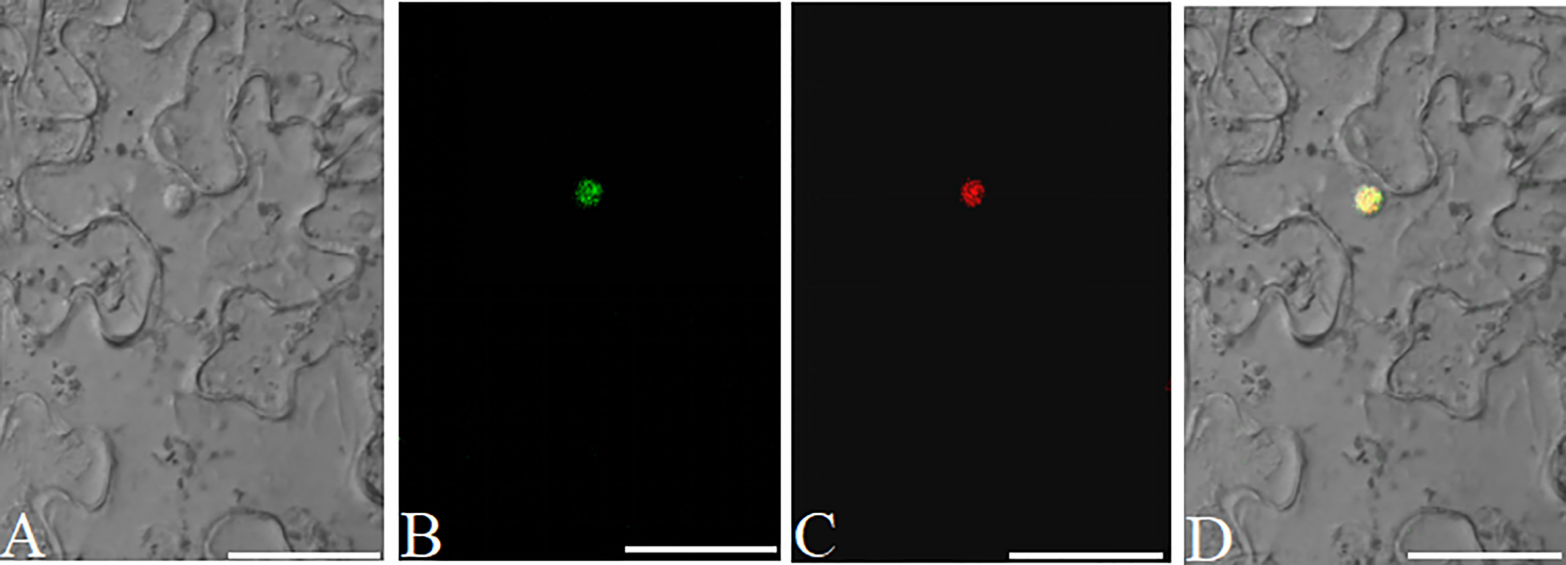
Analyses of the subcellular localization of VvCEB1_opt_ in *N. benthamiana* leaf cells using the pK35BTR2GFP vector. Bright field (BF) **(A)**. Fluorescent signal of VvCEB1opt-eGFP fusion protein **(B)**. Subcellular localization of nucleus markers using mCherry fluorescent protein (mChe) **(C)**; Merge: overlapping of the eGFP, mChe, and BF images **(D)**. Scale bars=50 μm.

Protein subcellular localization is crucial for studying plant gene function(s) and cellular biological processes. eGFP is a fluorescent marker commonly used for protein subcellular localization. We created two stable plant binary expression vectors, pK35BTR1/2GFP (*Kan*^R^), that allow C-terminal protein fusion with eGFP (**Figure 3**). A linker sequence that codes a peptide linkage (GGSGGS), 5’-GGTGGATCCGGAGGTTCT-3’, was designed and inserted between the candidate gene and *eGFP* gene (**Figure 3**) (Hao et al., 2005). To validate that the vectors were suitable for studying protein subcellular localization, we fused the *eGFP* in pK35BTR2GFP with the *Vitis vinifera VvCEB1_opt_*, which encodes a transcription factor (Nicolas et al., 2013; Lim et al., 2018). The GFP fluorescent signal was detected in the nucleus when fused with VvCEB1_opt_ (**Figure 8**), which is in agreement with localization of VvCEB1_opt_ in previous reports (Nicolas et al., 2013; Lim et al., 2018). The result indicated that the protein subcellular localization vectors are reliable.

### Generation of multiple expression vectors cloned with different FDIs by a single step digestion–ligation reaction

Genome-scale functional annotation of large numbers of candidate genes will be conducted in the post-genome era. A fast and highly efficient cloning method is the prerequisite for the functional annotation of such genes. Next, we tested whether several FDIs could be independently cloned into the pBTR vectors to generate multiple corresponding expression vectors carrying different FDIs by a single step digestion–ligation reaction (each expression vector cloned an FDI and no different FDIs stacked in one vector). For this purpose, four genes, *Glyma.02g025400* (3200 bp), *Glyma.05g201700* (2616 bp), *Glyma.06g165700* (2068 bp), and *Glyma.17g095000* (2852 bp), were cloned into pR35BTR2. Among these four genes, *Glyma.06g165700* carries a *Bsa*I recognition site. Ten white colonies were randomly selected after the digestion–ligation mixtures were transformed into competent *E*. *coli* cells. The plasmids were extracted and subjected to digesting by *Hin*dIII to identify the inserts (**Figure S3A**). The inserted fragments were further determined by Sanger sequencing (**Figure S3B**). All ten white colonies were positive transformants and carried the expected inserts. The recombinant frequencies of the four FDIs were as follows: *Glyma.02g025400* (2/10, 20%), *Glyma.05g201700* (3/10, 30%), *Glyma.06g165700* (2/10, 20%), and *Glyma.17g095000* (3/10, 30%). The result shows that the pBTR1/2 vectors can be used for multiple FDIs cloning to construct expression vectors by one-step digestion–ligation reaction in one tube by a single transformation.

## Discussion

### pBTR vectors: highly efficient, cost-efficient, and convenient plant binary expression vectors

Gene (over)expression analysis, promoter activity analysis, and protein subcellular localization are crucial for studying plant gene function(s) and cellular processes. In these analyses, the first step is the construction of expression vectors. To this end, Golden Gate cloning provides a rapid, inexpensive assembly of multi-component DNA fragments that can be inserted into a destination cloning vector (Engler et al., 2008). Lampropoulos et al. (2013) used this method to develop a GreenGate system that can rapidly and sequentially integrate multiple pre-made modules into a plant expression vector (added two *Bsa*I recognition sequences arranged in reverse orientation in multiple cloning sites) by one-step digestion–ligation reaction completed in one tube. Emami et al. (2013) developed three plant expression vectors based on Golden Gate cloning. However, only two plant selection markers/reporters can be used for plant transformation. In addition, expression-related cassettes, such as promoters and terminators, have not been pre-recombined into the expression vector. Multiple DNA fragments including the FDI need to be sequentially recombined into the expression vector. However, cloning efficiency significantly decreases with an increasing number of cloned modules.

In this study, based on the Golden Gate cloning method (Engler et al., 2008), we developed a series of novel pBTR vectors in which expression-related modules and several types of plant selection markers or reporter genes (e.g., *Kan^R^*, *Hyg^R^*, *Bar^R^*, and *DsRed*) have been previously cloned into the expression vectors (**Figures 1**–**3**). The diverse types of plant selection markers or reporter gene in the different pBTR vectors provide a convenient selection applied for different plant transformation events. The pBTR expression vectors require only a single FDI cloned into them, which is greatly convenient and highly efficient.

One limitation of Golden Gate cloning is to clone FDI carrying one or several internal restriction enzyme site(s), such as *Bsa*I used, into the expression vector. Engler et al. (2008) suggested three possible methods to bypass this problem. The first strategy is to run the digestion–ligation program, inactivate the *Bsa*I by heating, and then append new T4 ligase to the mixtures sequentially. This is efficiently validated (Engler et al., 2008; Emami et al., 2013), but it is a complex operation. In addition, the gel-purify step needs to be added in GreenGate system (Emami et al., 2013). The second strategy requires another set of expression cloning vectors that are different from the *Bsa*I enzyme used, which is not recommended by Engler et al. (2008). The third strategy is to eliminate any internal *Bsa*I restriction site(s) carried in the FDI, which is also time-consuming, complex, and laborious. In this study, using pBTR vectors and our modified digestion–ligation program, even FDI carries two internal *Bsa*I recognition sites (exemplified by cloning of *GUSPlus* and *GmbHLH293*), which can also be highly efficiently cloned into the pBTR vector using our one-step digestion–ligation program (**Figure 4**). In our modified digestion–ligation program, inactivation of *Bsa*I *via* heating and re-supplement of fresh ligase to the digestion–ligation mixtures are not required. FDI carrying one, or more recognition site(s) is to be cloned into the pBTR vector, in that case, after running the program of cycles, the final incubation temperature is set at 4°C for 1 h in order to religate the linearized FDI by *Bsa*I. Hence, it is a simple operation to clone a single FDI carrying internal *Bsa*I site(s) into the expression vector.

A DNA molecule carrying *Bsa*I site(s) would produce a 4-nt overhang after digestion by *Bsa*I; thus, theoretically, 256 (4^4^) different sticky ends can be produced. *Bsa*I recognizes six base pair recognition sequences and cuts the DNA molecule outside its recognition sequences at the 3’ terminus. Restriction sites are expected to be present on average every 4, 096 bp (4^6^) in genomes. It is greatly low frequency to present the same sticky ends produced between an FDI (internal *Bsa*I recognition site) and the pBTR vectors (4^10^=1,048,576) because only a single FDI was cloned. In contrast, in expression vectors based on the GreenGate system, internal *Bsa*I sites carried by FDIs can complicate cloning because digestion at such sites might generate the same sticky end sequences, thereby greatly decreasing the correctness and integrity of multi-component DNA fragments into the destination vector. In this study, to avoid this issue, we developed two sets of pBTR vector, each of which carried a different neighbor sequence flanked by *Bsa*I recognition sites and would thus generate different sticky ends. If a particular FDI with a *Bsa*I recognition sequence has the same sticky end as that of the pBTR1 set, in such case, the pBTR2 set can be used. That is, any two sets of pBTR vector will be suitable for almost all FDIs and are also sequence-independent.

We modified the digestion–ligation procedure of Engler et al. (2008) as more time cycles replacing a single digestion–ligation reaction. In our procedure, in each cycle, the FDI and original pBTR vector are digested by *Bsa*I at 37°C for 3 min, then the completely digested FDI and pBTR vector are recombined as a circular DNA at 16°C for 5 min; at which point another gradient ligation at 12°C for 2 min is run. In the subsequent cycles, if the FDI is cloned into the original destination pBTR vector, the *Bsa*I recognition sequences in that vector would be eliminated in the novel recombinant DNAs. Therefore, it would not be possible to re-cut these DNAs in subsequent cycles. The left non-recombinant original pBTR vector would continue to be cut over and over again by *Bsa*I in the following digestion steps. It doesn’t be re-digested in the digestion step after FDI is recombined into the pBTR vector. This creates a one-way digestion–ligation reaction in one tube. Even FDI carries internal *Bsa*I recognition site(s), the digested FDI caused in the digestion step can be re-ligated into an integrated DNA molecular again in the final ligation step at 4°C for 1 h. Therefore, the FDI with internal *Bsa*I recognition site(s) can also efficiently be cloned into the pBTR vector (different overhangs at 5’-end and 3’-end with the vector). Furthermore, these vectors can be used to clone multiple FDIs to construct corresponding multiple expression vectors in a single-step digestion–ligation and transformation event. Hence, the pBTR vectors are cost-efficient, easy-to-use, and highly efficient.

A marker or reporter gene is often used to facilitate the screening of positive recombinants in *E*. *coli*. To this end, antibiotic genes such as *lac-Z* or *ccdb* are commonly used (Xing et al., 2014; Ma et al., 2015). However, this requires additional chemical reagents. Emami et al. (2013) developed three plant expression vectors based on Golden Gate cloning using *lac-Z* as a selective marker in *E*. *coli*, which requires the chemical reagent X-gal. The fluorescent proteins, such as GFP, YFP, RFP, CFP, etc., are often used as a reporter in screening of transgenic plants in various plant species (Fan et al., 2020b). However, to date, no such expression vector carrying fluorescent protein has been found and be used as an indicator in *E*. *coli* to select recombinant colonies by the unaided eye. Recently, it was reported that mScarlet-I is a monomeric red fluorescent protein with fast maturity time and high brightness (Bindels et al., 2017; Mccullock et al., 2020). In this study, we developed pBTR vectors carrying *mScarlet-Ⅰ* to enable to visualize recombinant colonies by the naked eye without a need for additional substrate/reagents or special *E*. *coli* strains. This is greatly convenient for screening positive colonies. Additionally, to facilitate the user to recombine expression-related modules or special tags with flexibility due to special destination or designation in need, we also developed a set of p-SBTR1/2 vectors with a visual mScarlet-I selection marker that can meet the requirements (**Figure S4**).

### Some crucial steps to consider when using the pBTR vectors

To achieve directional cloning, special adapters added at the 5’-end of primers for different destination vectors should be selected carefully (listed in **Table 1**). To clone an FDI into the pBTR vectors, for one thing, as for no internal *Bsa*I recognition site(s) presented in the FDI, both pBTR1 and pBTR2 sets can be used. For another, one or more internal *Bsa*I site(s) presented in the FDI, the same sticky ends produced by *Bsa*I between FDI and vector should be avoided. In the digestion–ligation reaction program (**Figure 4**), the final incubation temperature is held at 4°C for 1 h. This is done with the aim to religate the linearized FDI (with internal *Bsa*I sites) by *Bsa*I. Generally, the molecular molar ratio of FDI to vector should be 1:1 when no internal *Bsa*I site(s) is carried by the FDI or when different non-palindromic sticky ends are produced with internal *Bsa*I site(s). We recommend that the molar ratio of foreign DNA to vector should be 3–5:1 for palindrome sticky end(s) generated from FDI. To reduce costs without sacrificing *Bsa*I digestion efficiency, we recommend adding four protective bases to the 5’ terminus of primers.

## Conclusion

A novel series of plant binary expression vectors, termed pBTR vectors, were developed. The pBTR vectors were used with a highly simplified cloning step, single *Bsa*I restriction enzyme required, DNA purification-free, almost sequence-independent, directional cloning, multiple target genes cloning, cost-effective, and directly visual selection marker for screening positive recombinants colonies with the unaided eye. We anticipate that these vectors will be widely used for analyses of gene (over)expression, promoter activity, and protein subcellular localization. These vectors will promote functional genomics research in plants and are free for users by non-profit researchers.

## Methods

### Plant materials and growth conditions

Seeds of soybean (*Glycine max* cv. Williams82), tomato (*Solanum lycopersicum*, local variety Maofen802) , *Arabidopsis thaliana* Columbia (Col-0), and transgenic *Nicotiana benthamiana* were kept in our lab. The *N. benthamiana* was transformed with a mCherry fluorescent protein marker fused with nuclear localization peptide signal of SV40 (Simian virus 40) large T antigen (van der Krol and Chua, 1991). All plants were cultivated in a growth chamber (24–26°C, 16 h light/ 8 h dark cycle).

### Construction of gene (over)expression vectors p-35BTR1/2, p-UbiBTR1/2, and p-NatBTR1/2

To construct p-35BTR1/2 and p-UbiBTR1/2, the CRISPR/Cas9 vectors pHSE401/pKSE401 and pYLCRISPR/Cas9Pubi-H/B/N (Ma et al., 2015) were used. To remove the sequences between the two *Hin*dIII sites (including two *Bsa*I recognition sequences, the SpR-gRNA-scaffold, and the expression cassette of SmR), the pHSE401/pKSE401 vectors were digested with *Hin*dIII at 37°C for 30 min in 1×T4 ligation buffer followed by 65°C for 15 min (*Hin*dIII inactivated), and then 10 U T4 ligase was added to the reaction mixtures and held at 16°C for 1 h to religate the vectors. The digestion–ligation mixtures were transformed into chemically competent *Trelief*^TM^5α (>10^10^cfu/ug, Tsingke Biotech Co., Ltd., Qingdao, China) *E*. *coli* cells *via* heat shock at 42°C for 60 s. The two generated vectors, in which 1919 bp sequences between the two *Hin*dIII sites were deleted (primary regions 268–2187 bp in pHSE401 [Addgene Plasmid #62201], and 268–2187 bp in pKSE401 [Addgene Plasmid #62202]; Xing et al., 2014), were termed pH35Cas9 and pK35Cas9, respectively. To construct the pH35BTR1/pK35BTR1 and pH35BTR2/pK35BTR2 vectors (**Figure 1A**), the *Cas9* gene was replaced with the cassette of P*_nptⅡ::_ mScarlet-I* based on pH35Cas9 and pK35Cas9. The *nptII* promoter (P*_nptⅡ_*) and *mScarlet-I* coding sequences were amplified *via* PCR with two primer sets, mSXb1F/mSSa1R and mSXb2F/mSSa2R, respectively, using pMRE-Tn5-155 (Addgene Plasmid #118537) (Schlechter et al., 2018) as the template. The PCR products were digested using restriction enzymes *Xba*I and *Sac*I for cloning into pH35Cas9 and pK35Cas9 that were previously digested by *Xba*I and *Sac*I, thereby producing pH35BTR1/pK35BTR1 and pH35BTR2/pK35BTR2, respectively (**Figure 1A**).

To construct vectors p-35BTR1/2 with various selected markers, a *DsRed* gene was used to replace the *hygromycin*-resistance gene in the pH35BTR1 and pH35BTR2 vectors. *DsRed* (from pRed1305 plasmid; Fan et al., 2020c) was cloned into pH35BTR1 and pH35BTR2 between the *Eco*RI and *Sac*II restriction sites using *Eco*RI and *Sac*II digestion, respectively, thereby generating pR35BTR1 and pR35BTR2 (**Figure 1A**).

To construct p-UbiBTR1/2 (**Figure 1B**), pYLCRISPR/Cas9Pubi-H/B/N backbones were used (Ma et al., 2014). The *mScarlet* gene and *E9* terminator cassettes were amplified *via* PCR with primer sets mSYL1/2 and mSYL2/3 using plasmids pR35BTR1 and pR35BTR2 as the templates with the High-Fidelity DNA Polymerase Enzyme KOD-plus kit (TOYOBO Biotech, Osaka, Japan), respectively. The two PCR products were digested with *Pst*I for cloning into pYLCRISPR/Cas9Pubi-H/B/N that was previously digested by *Pst*I and *Pme*I. These recombinant vectors were termed as pHUbiBTR1/2, pBUbiBTR1/2, and pKUbiBTR1/2, respectively (**Figure 1B**).

To construct expression vectors p-NatBTR1/2 (**Figure 1C**) driven by the native promoter, the *mScarlet-I* expression cassette was amplified by PCR with two primer sets, mSHin3/mSSa1R and mSHi32/mSSa4R, respectively, using pMRE-Tn5-155 as the template. The PCR products were digested with *Hin*dIII and *Sac*I for cloning into pH35Cas9, pK35Cas9, and pR35BTR1 that were previously digested with *Hin*dIII and *Sac*I, respectively, creating the expression vectors pHNatBTR1/2, pKNatBTR1/2, and pRNatBTR1/2 (**Figure 1C**).

To construct the pK35BTR1*-AtMyb75*, pK35BTR2*-GUSPlus*, and pR35BTR2-*GmbHLH293* vectors, *AtMyb75*, *GUSPlus*, and *GmbHLH293* were amplified *via* PCR with the primers At75BF/At75BR (for *AtMyb75*), GUS1F/GUS1R (for *GUSPlus*), and Gmb293F/Gmb293R (for *GmbHLH293*), using p35SAt75 plasmid (Fan et al., 2020b), pCAMBIA1305.1 plasmid, and Williams82 genomic DNA as templates, respectively. The PCR products were digested with *Bsa*I for cloning into pK35BTR1, pK35BTR2, and pR35BTR2, thereby generating pK35BTR1*-AtMyb75*, pK35BTR2*-GUSPlus*, and pR35BTR2-*GmbHLH293* vectors, respectively. The one-step restriction digestion–ligation reaction was performed as follows. Vector p-35BTR1/2 (~20 ng), PCR products (~20 ng), *Bsa*I (10U, New England Biolabs, Ipswich, MA, USA, #R3535L), T4 ligase (10U, New England Biolabs, Ipswich, MA, USA, #M0202L), and 1×NEB rCutsmart buffer were mixed with 1mM ATP in one tube. The one-step restriction digestion–ligation reaction was performed in a thermo-cycler as follows: 10 cycles at 37°C for 3 min, at 16°C for 3 min, and at 12°C for 2 min, followed by a final incubation step at 4°C for 1 h (to clone *GUSPlus* and *GmbHLH293* into pK35BTR2 and pR35BTR2) or at 37°C for 10 min (to clone *AtMyb75* into pK35BTR1). Subsequently, the digestion–ligation mixtures were transformed into competent *Trelief*^TM^5α. The white colonies were selected to identify the positive recombinants. The recombinant plasmids were further tested using *Hin*dIII and *Xba*I (for pK35BTR1*-AtMyb75*) and *Hin*dIII (for pK35BTR2*-GUSPlus* and pR35BTR2-*GmbHLH293*), and Sanger sequencing, respectively.

### Construction of promoter analysis vectors pKBTR1/2PGUS, pHBTR1/2PGUS, and pRBTR1/2PGUS

To construct pKBTR1/2PGUS, pHBTR1/2PGUS, and pRBTR1/2PGUS (**Figure 2**), *GUSPlus* gene (without internal *Bsa*I recognition site) was used to replace the expression cassette P*_nptⅡ_*::*mScarlet-I* in the pH35BTR1 vector, following the protocol described in **Figure 4**. *GUSPlus* was amplified *via* PCR using the primers GUS2F and GUS2R with pGSE401 plasmid (Fan et al., 2020a) as a template, generating the intermediate vectors pH35GUS. The expression cassette of P*nptⅡ*::mScarlet-I was amplified with primers mSHin3 and mSXba1(to produce pHBTR1G), and primers mSHi32 and mSXb12 (to produce pHBTR2G), using pR35BTR2 as a template. The PCR products were digested with *Hin*dIII and *Xba*I and directly ligated into pH35GUS, which were previously digested with *Hin*dIII and *Xba*I, and produced two mediate vectors, pHBTR1G and pHBTR2G, respectively.

Because the 35S enhancer sequences presented in pHBTR1G and pHBTR2G vectors may influence the expression activity of nearby promoters, we used the double 35S to drive the expression of selection reporter gene/marker (*Hyg^R^*, *Kan^R^*, and *DsRed*) and replaced the enhanced CaMV35S promoter sequences in the pHBTR1G and pHBTR2G. Two oligos DNA, MCS41 and MCS42, were chemically synthesized and annealed at 65°C for 5 min. Then the annealed DNA fragment was inserted into the site of *Hin*dIII of pH35BTR1, and thus produced the new intermediate vector pH35BTR1MCS, which was introduced a restriction endonuclease site of *Eco*RI. Three primer sets, HygS1 and HygS2, KanS1 and KanS2, RedS1 and RedS2, were used to amplify *Hyg^R^*, *Kan^R^*, and *DsRed* gene, respectively. The three responding PCR products were mixed with pH35BTR1MCS added with *Xho*I, *Sal*I, and T4 DNA ligase in 1×NEB rCutsmart buffer (with 1 mM ATP), respectively. The one-step digestion–ligation reaction was performed as follows: 10 cycles at 37°C for 3 min, at 16°C for 3 min, and at 12°C for 2 min, followed by at 37°C for 10 min. The generated three new vectors were named p35HygM, p35KanM, and p35RedM, respectively. The pHBTR1G and pHBTR2G were digested with *Hin*dIII and *Eco*RI, generated a fragment of 3846-bp and 3851-bp, respectively, and introduced into between *Hin*dIII and *Eco*RI of p35HygM, p35KanM, and p35RedM, and produced the corresponding promoter analysis vectors, pHBTR1/2PGUS, pKBTR1/2PGUS, and pRBTR1/2PGUS produced (**Figure 2**).

To construct pRBTR1PGUS-*AtGCS*, a 1178-bp upstream promoter region of the translation start site of the gamma-*glutamylcysteine synthetase* gene (GenBank accession no. AF068299.1) was amplified *via* PCR using primers AtGa4F and AtGa4R from *A. thaliana* Columbia (Col-0) genomic DNA as a template. The PCR products were cloned into pRBTR1PGUS using the protocol described in **Figure 4** in this study.

### Construction and verification of subcellular localization vectors

To construct subcellular localization vectors, pK35BTR1/2 was used as a backbone. The *eGFP* was amplified *via* PCR with a forward primer GFPB1 carrying a linker sequence, GGTGGATCCGGAGGTTCT, that codes a peptide linkage (GGSGGS) and a reverse primer GFPB2, using pNC-TRV2-GFP plasmid as a template (Yan et al., 2020). The *eGFP* gene was used to replace the expression cassette P*_nptⅡ_*::*mScarlet-I* in the pK35BTR1/2, generating the intermediate vector pKG according to the protocol in **Figure 4**. To introduce the expression cassette P*_nptⅡ_*::*mScarlet-I* into the pKG and produce the corresponding subcellular localization vector pK35BTR1/2GFP, the expression cassette P*_nptⅡ_*::*mScarlet-I* was amplified with primers mSXb1F/mSBam1 (to produce pK35BTR1GFP) and mSXb2F/mSBam2 (to produce pK35BTR2GFP), using pMRE-Tn5-155 plasmid as a template (Schlechter et al., 2018). The PCR products were digested with *Bam*HI and *Xba*I, and directly ligated into pKG that was previously digested with *Bam*HI and *Xba*I, thus producing pK35BTR1/2GFP (**Figure 3**).

The *VvCEB1_opt_* gene (Nicolas et al., 2013; Lim et al., 2018) was used to verify pK35BTR2GFP. *VvCEB1_opt_* without the stop codon was cloned into pK35BTR2GFP between the two *Bsa*I cleavage sites to generate the pK35*VvCEB*-GFP fusion vector. *VvCEB1_opt_* was amplified *via* PCR with primers SuVvCF and SuVvCR from synthetic *VvCEB1_opt_* cDNA (kept in our lab) as a template. The digestion–ligation reaction was incubated for 10 cycles (37°C, 3 min; 16°C, 5 min; 12°C, 2 min) and then stayed at 37°C for 2 min (*VvCEB1_opt_* carrying no *Bsa*I site).

### Construction of binary expression vectors p-SBTR1/2 carrying no expression-related modules

Two PCR products were amplified with pR35BTR1 as a template by primers sets, mSHin3 and mSEco1, mSHi32 and mSEco2 (**Table S1**), and then were digested with *Hin*dIII and *Eco*RI for cloning into the same restriction sites of vectors, pK35Cas9, pH35Cas9, and pR35BTR1, respectively. The three newly generated vectors were named pSKBTR1/2, pSHBTR1/2 and pSRBTR1/2 (**Figure S4**).

### Multiple fragments cloned into the pR35BTR2 to construct multiple expression vectors

To verify whether the pBTR1/2 vectors are suitable for constructing multiple expression vectors carrying different FDIs in a single reaction in a single transformation, four genes, *Glyma.02g025400*, *Glyma.05g201700*, *Glyma.06g165700*, and *Glyma.17g095000*, were cloned into pR35BTR2 according to the protocol described in **Figure 4**. The four genes were amplified using primers Gm2c4F/Gm2c4R, Gm5c7F/Gm5c7R, Gm6c7F/Gm6c7R, and Gm17c5F/Gm17c5R by PCR, respectively. The four PCR products were mixed (molar ratio=1:1:1:1) together with the pR35BTR2 vector (FDI: vector molar ratio=1:1). The digestion–ligation reaction was performed according to the protocol in **Figure 4** with 10 cycles (37°C, 3 min; 16°C, 5 min; 12°C, 2 min) and then a holding period at 4°C for 1 h. All primers used in this study are listed in **Table S1**. All constructs were confirmed by Sanger sequencing. Entire sequences of all the pBTR vectors constructed in this paper are given in **Supplementary Material Table S2** and the plasmids will be available *via* Addgene (https://bccm.belspo.be/deposit/public/plasmids).

### Hairy root transformation mediated by *A. rhizogenes* and *Arabidopsis* transformation mediated by *A. tumefaciens*

The vectors pK35BTR1*-AtMyb75* and pK35BTR2*-GUSPlus* were introduced into *A. rhizogenes* strain K599 by electroporation. Composite soybean and tomato plants were generated by one-step *A. rhizogenes*-mediated transformation as published previously (Fan et al., 2020a) with minor modification. To inoculate the K599 harboring the plasmid of interest, the step of watering the K599 with the transformed plasmid was removed. The pRBTR2PGUS-*AtGCS* construct described above was introduced into wild-type *A. thaliana* plants using the floral dip method (Clough and Bent, 1998).

### GUS staining and subcellular localization assays

Histochemical staining of GUS activity assay followed the protocol (Jefferson et al., 1987; Fan et al., 2017; Fan et al., 2020c). Plant tissues materials were stained in the GUS staining solution (100 mM sodium phosphate at pH 7.0, 0.1% Triton X-100, 1 mg/mL X-Gluc, 1 mM potassium ferricyanide, and 1 mM potassium ferrocyanide) in the dark at 37°C for 5-10 h. The roots stained were rinsed in 70% ethanol for 10-20 min. Transient expression with subcellular localization vector was analyzed in the transgenic *N. benthamiana* following the protocol (Sparkes et al., 2006). pK35*VvCEB*-GFP was transformed into *A. tumefaciens* strain GV3101 by electroporation. The GFP fluorescence of tobacco leaves was observed and photographed using a confocal laser scanning microscope (Zeiss LSM 700, Germany) with a Fluar ×10/0.50 M27 objective lens and SP640 filter after 48 h cultivation (Carl Zeiss).

### Reverse transcription-polymerase chain reaction analysis

The total RNA was isolated, first-strand cDNA was synthesized, and RT-PCR amplification was performed as previously described (Lü et al., 2010; Fan et al., 2020a). To analyze the expression of *GUSPlus* and *AtMYB75* in the transgenic hairy roots of tomato and soybean transformed with pK35BTR1*-AtMyb75* and pK35BTR2*-GUSPlus*, RT-PCR reaction was carried out in a reaction mixture to amplify the endogenous gene and *AtMYB75* (tomato roots transformed with pK35BTR1*-AtMyb75*) or *GUSPlus* (soybean roots transformed with pK35BTR2*-GUSPlus*), respectively. The tomato *SlEF* (Solyc06g005060) (Liao et al., 2015) and soybean endogenous gene *GmActin* (Fan et al., 2020a) were used as a endogenous gene control, respectively. To analysis the transcript of *GUSPlus* in the 7-day-old seedlings of *A*. *thaliania* transformed with pRBTR1PGUS-*AtGCS*, RT-PCR reaction was performed in a reaction mixture to amplify the *A*. *thaliania* endogenous gene *ACTIN* (At3g18780) and exogenous *GUSPlus*. The gene-specific primer pairs SlEFF and SlEFR for *SlEF* (Liao et al., 2015), GmActinF and GmActinR for *GmActin* (Fan et al., 2020a), AtActinF and AtActinR for *A*. *thaliania* endogenous gene *ACTIN*, RTGusF and RTGusR for *GUSPlus* (Fan et al., 2020a), At751 and At752 for *AtMyb75* were used. RT-PCR experiments were performed with three replicates. All primers sequences used in this paper are listed in **Supplemental Table S1**.

## Data availability statement

The original contributions presented in the study are included in the article/**Supplementary Material**. Further inquiries can be directed to the corresponding authors.

## Author contributions

YF and SHL designed the experiments and wrote the paper. XW, CT, HW, SL, HX, WP, QL, HH, and QYL performed the research work. All authors contributed to the article and approved the submitted version.

## Supplementary material

Supplementary Figure 1 Verification of positive recombinants in *E*. *coli* transformed with pR35BTR2-*GmbHLH293* and analysis of cloning efficiency. Electrophoretogram of enzyme digestion of pR35BTR2-*GmbHLH293* recombinant plasmids with *Hin*dIII. M, DL5000 DNA marker (bought from Tsingke Co., Qingdao, China); Lane 1, pR35BTR2 plasmid used as a control; Lanes 2–9, eight randomly selected pR35BTR2-*GmbHLH293* plasmids isolated from white *E*. *coli* colonies **(A)**. An example of Sanger sequencing analysis to verify the presence of *GmbHLH293* fragment in the pR35BTR2 vector (partial sequences shown) **(B)**. Analysis of cloning efficiency in *E*. *coli* transformed with pK35BTR1*-AtMyb75* **(C)** and pK35BTR2*-GUSPlus* **(D)**.

Supplementary Figure 2 RT-PCR assays on transgenic hairy roots/plants expressing *AtMyb75*/*PAP1* or *GUS*. Expression of *AtMyb75*/*PAP1* in transgenic hairy roots. Lanes 1-2, No Purple/red colored anthocyanin accumulated on non-transgenic root inoculated K599 with pK35BTR1*-AtMyb75* plasmid; Lane 3, pK35BTR1*-AtMyb75* plasmid; Lanes 4-8, Purple/red colored anthocyanin accumulated on independent transgenic root inoculated K599 with pK35BTR1*-AtMyb75* plasmid; Lane 9, *A*. *rhizogenesis* K599; Lane 10, ddH_2_O **(A)**; RT-PCR analysis on transgenic hairy roots expressing *GUSPlus*. Lanes 1-2, GUS-negative non-transgenic root inoculated K599 with pK35BTR2-*GUSPlus* plasmid; Lane 3, pK35BTR2-*GUSPlus* plasmid; Lanes 4-8, GUS-positive independent transgenic root inoculated K599 with pK35BTR2-*GUSPlus* plasmid; Lane 9, *A*. *rhizogenesis* K599; Lane 10, ddH_2_O **(B)**; RT-PCR analysis on *GUSPlus* transgene expression in the *A. thaliana* plants transformed with pRBTR1PGUS-*AtGCS*. Lane 1-2, ddH_2_O; Lanes 3-7, Independent GUS-positive transgenic *A*. *thaliana* line; Lane 8, GUS-negative transgenic *A*. *thaliana* line; Lane 9, pRBTR1PGUS-*AtGCS* plasmid **(C)**.

Supplementary Figure 3 Validation of multiple fragments cloned into the pR35BTR2 expression vector. Electrophoretogram of enzyme digestion of plasmid DNA extracted from 10 white colonies digested with *Hin*dIII. M, DL5000 DNA marker; Lanes 1, 8, pR35BTR2-*Glyma.06g165700*; Lanes 2, 5, 7, pR35BTR2-*Glyma.05g201700*; Lanes 3, 6, 10, pR35BTR2-*Glyma.17g095000*; Lanes 4, 9, pR35BTR2-*Glyma.02g025400* **(A)**. Examples of Sanger sequencing analyses of candidate FDIs cloned into the pR35BTR2 expression vector **(B)**.

Supplementary Figure 4 Diagram of p-SBTR1/2 vectors.
