# Supplementary figures and images for: Development of a set of novel binary expression vectors for plant gene function analysis and genetic transformation
Source: Front Plant Sci. 2023 Jan 12;13:1104905. doi: 10.3389/fpls.2022.1104905 (PMC9877630; doi:10.3389/fpls.2022.1104905)

**FIGURE S1**

A

**100 bp**


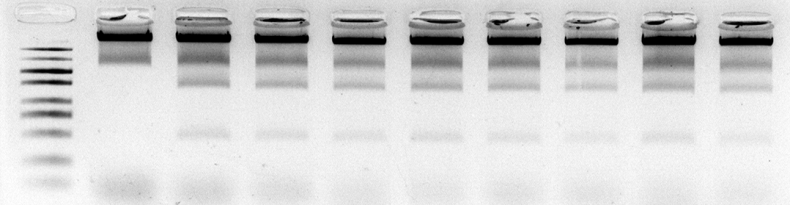


**M**

**1**

**2**

**3**

**4**

**5**

**6**

**7**

**8**

**9**

**250 bp**

**500 bp**

**750 bp**

**1000 bp**

**1500 bp**

**2000 bp**

**3000 bp**

**5000 bp**

B


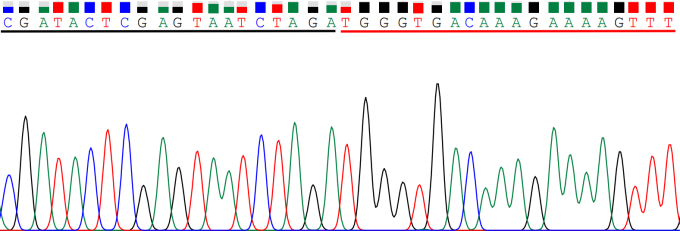


partial pR35BTR2 vector

partial *GmbHLH293* sequences


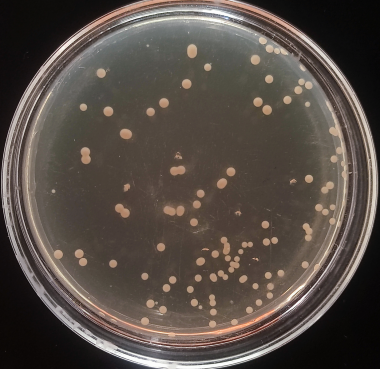
C

D


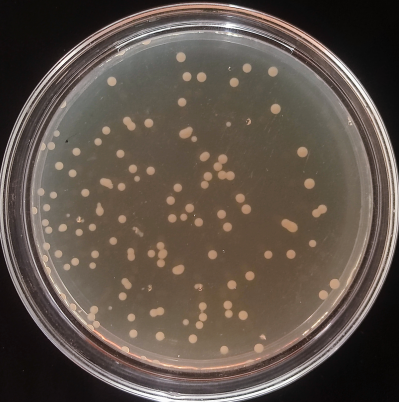

Supplement: Supplementary Figure 1 — Verification of positive recombinants in E. coli transformed with pR35BTR2-GmbHLH293 and analysis of cloning efficiency. Electrophoretogram of enzyme digestion of pR35BTR2-GmbHLH293 recombinant plasmids with HindIII. M, DL5000 DNA marker (bought from Tsingke Co., Qingdao, China); Lane 1, pR35BTR2 plasmid used as a control; Lanes 2–9, eight randomly selected pR35BTR2-GmbHLH293 plasmids isolated from white E. coli colonies (A). An example of Sanger sequencing analysis to verify the presence of GmbHLH293 fragment in the pR35BTR2 vector (partial sequences shown) (B). Analysis of cloning efficiency in E. coli transformed with pK35BTR1-AtMyb75 (C) and pK35BTR2-GUSPlus (D). [file DataSheet_1.docx]

**FIGURE S4**


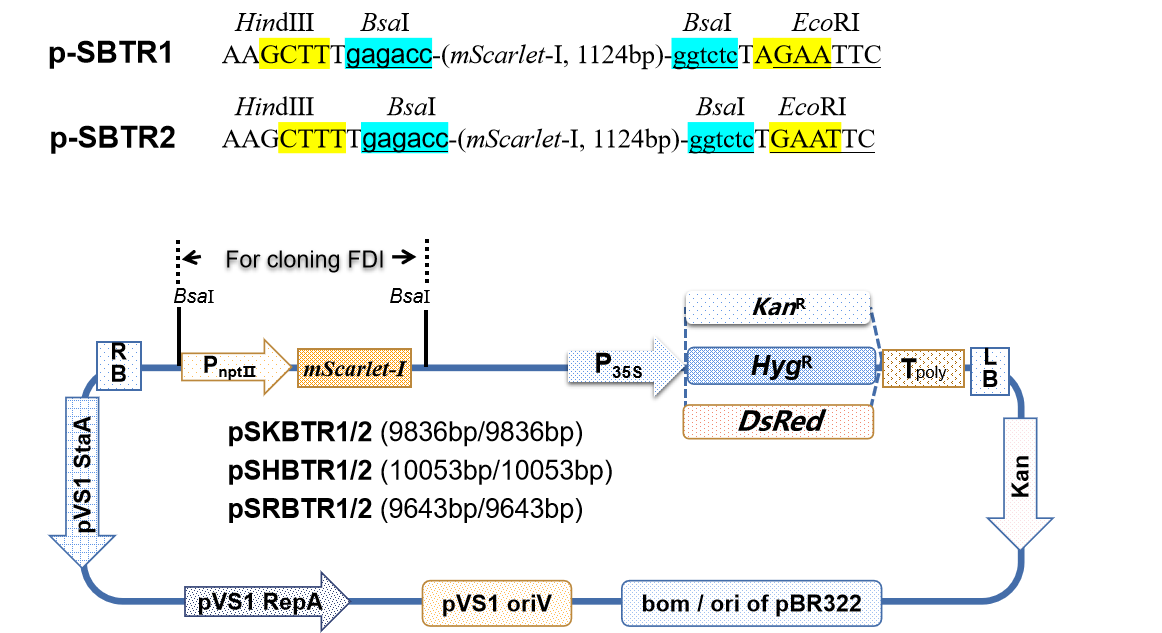

Supplement: Supplementary Figure 4 — Diagram of p-SBTR1/2 vectors. [file DataSheet_4.docx]
