# Supplementary material for: Development of a set of novel binary expression vectors for plant gene function analysis and genetic transformation
Source: Front Plant Sci. 2023 Jan 12;13:1104905. doi: 10.3389/fpls.2022.1104905 (PMC9877630; doi:10.3389/fpls.2022.1104905)
Supplement: Supplementary Figure 2 — RT-PCR assays on transgenic hairy roots/plants expressing AtMyb75/PAP1 or GUS. Expression of AtMyb75/PAP1 in transgenic hairy roots. Lanes 1-2, No Purple/red colored anthocyanin accumulated on non-transgenic root inoculated K599 with pK35BTR1-AtMyb75 plasmid; Lane 3, pK35BTR1-AtMyb75 plasmid; Lanes 4-8, Purple/red colored anthocyanin accumulated on independent transgenic root inoculated K599 with pK35BTR1-AtMyb75 plasmid; Lane 9, A. rhizogenesis K599; Lane 10, ddH2O (A); RT-PCR analysis on transgenic hairy roots expressing GUSPlus. Lanes 1-2, GUS-negative non-transgenic root inoculated K599 with pK35BTR2-GUSPlus plasmid; Lane 3, pK35BTR2-GUSPlus plasmid; Lanes 4-8, GUS-positive independent transgenic root inoculated K599 with pK35BTR2-GUSPlus plasmid; Lane 9, A. rhizogenesis K599; Lane 10, ddH2O (B); RT-PCR analysis on GUSPlus transgene expression in the A. thaliana plants transformed with pRBTR1PGUS-AtGCS. Lane 1-2, ddH2O; Lanes 3-7, Independent GUS-positive transgenic A. thaliana line; Lane 8, GUS-negative transgenic A. thaliana line; Lane 9, pRBTR1PGUS-AtGCS plasmid (C). [file DataSheet_2.docx]

**FIGURE S2**

A

***SlEF***

***AtMYB75***

**1**

**2**

**3**

**4**

**5**

**6**

**7**

**8**

**9**

**10**

**M**

**100 bp**

**250 bp**

**500 bp**

**750 bp**

**1000 bp**

**2000 bp**


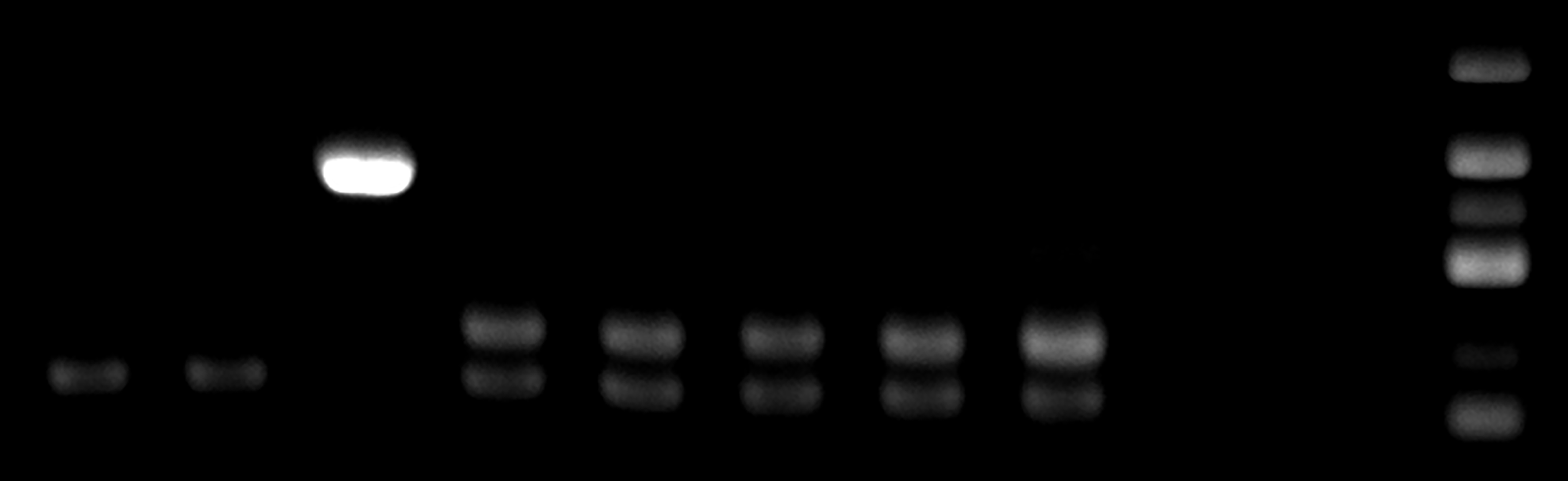


B


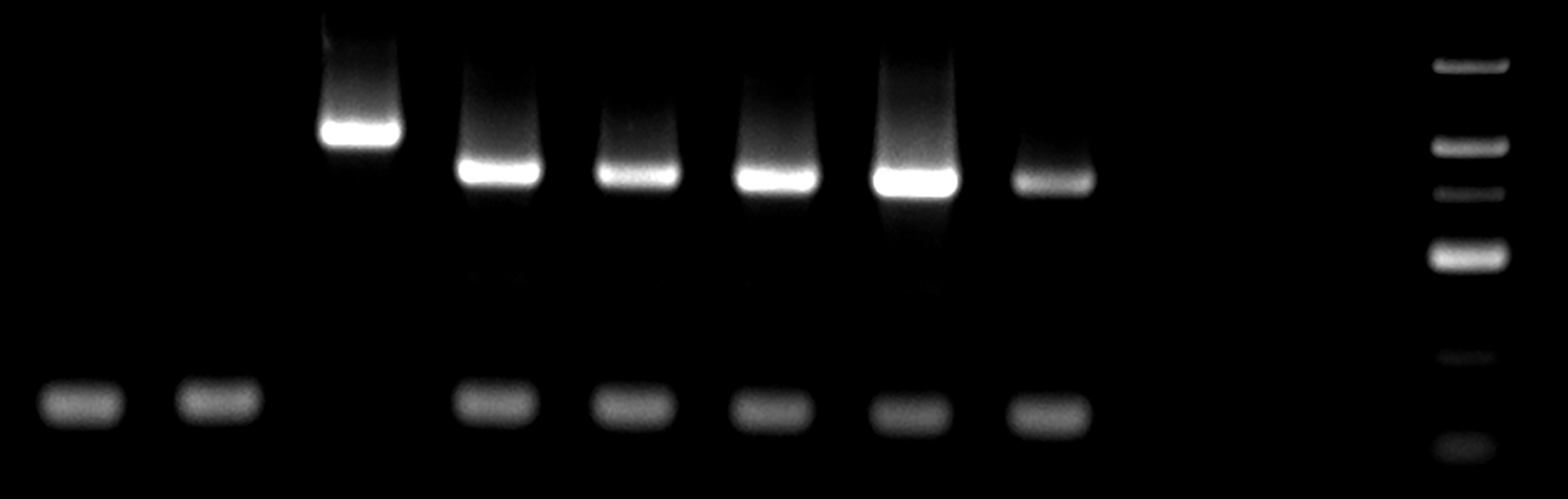


**100 bp**

**M**

**1**

**2**

**3**

**4**

**5**

**6**

**7**

**8**

**9**

**250 bp**

**500 bp**

**750 bp**

**1000 bp**

**2000 bp**

**10**

***GUSPlus***

***GmActin***


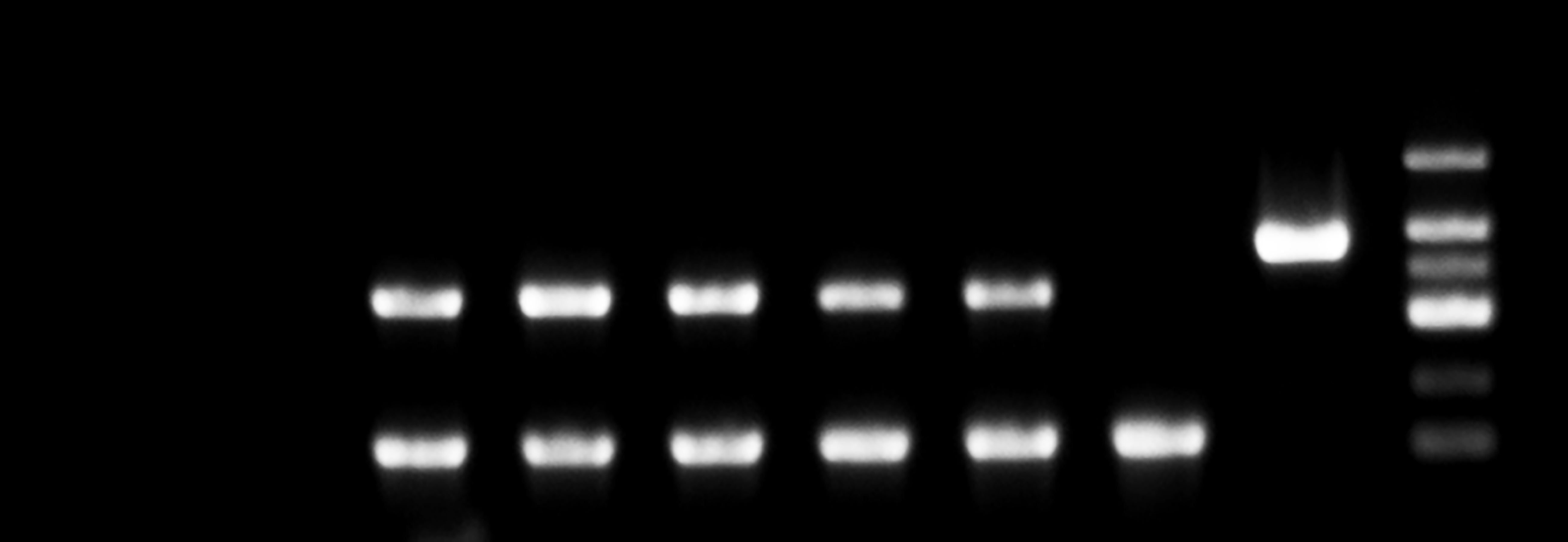
 C

**100 bp**

**M**

**1**

**2**

**3**

**4**

**5**

**6**

**7**

**8**

**250 bp**

**500 bp**

**750 bp**

**1000 bp**

**2000 bp**

**9**

***GUSPlus***

***Actin***
