# Supplementary material for: Development of a set of novel binary expression vectors for plant gene function analysis and genetic transformation
Source: Front Plant Sci. 2023 Jan 12;13:1104905. doi: 10.3389/fpls.2022.1104905 (PMC9877630; doi:10.3389/fpls.2022.1104905)
Supplement: Supplementary Figure 3 — Validation of multiple fragments cloned into the pR35BTR2 expression vector. Electrophoretogram of enzyme digestion of plasmid DNA extracted from 10 white colonies digested with HindIII. M, DL5000 DNA marker; Lanes 1, 8, pR35BTR2-Glyma.06g165700; Lanes 2, 5, 7, pR35BTR2-Glyma.05g201700; Lanes 3, 6, 10, pR35BTR2-Glyma.17g095000; Lanes 4, 9, pR35BTR2-Glyma.02g025400 (A). Examples of Sanger sequencing analyses of candidate FDIs cloned into the pR35BTR2 expression vector (B). [file DataSheet_3.docx]

**FIGURE S3**

A

**5000 bp**


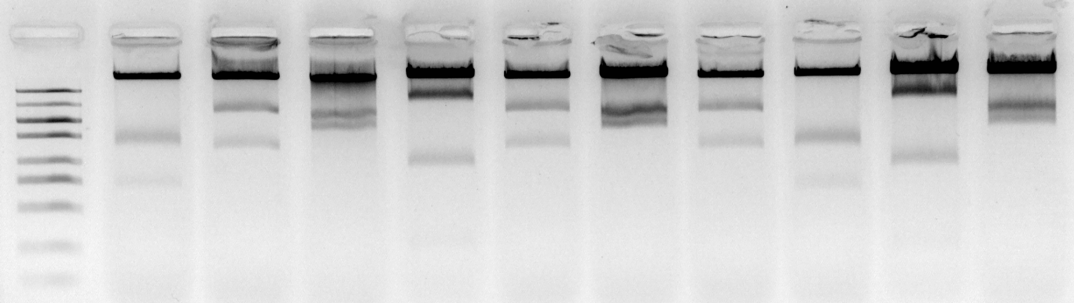


**100 bp**

**M**

**1**

**2**

**3**

**4**

**5**

**6**

**7**

**9**

**10**

**250 bp**

**500 bp**

**750 bp**

**1000 bp**

**1500 bp**

**2000 bp**

**3000 bp**

**8**

B


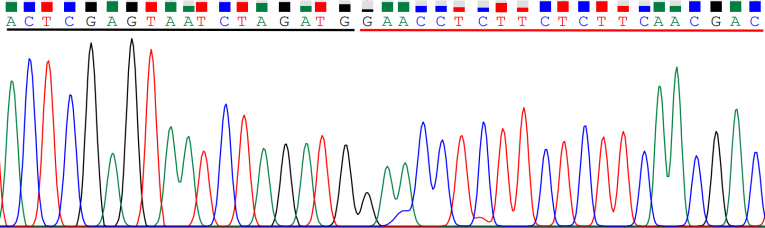


partial pR35BTR2 vector

partial *Glyma.02g025400* sequences

pR35BTR2-*Glyma.02g025400*


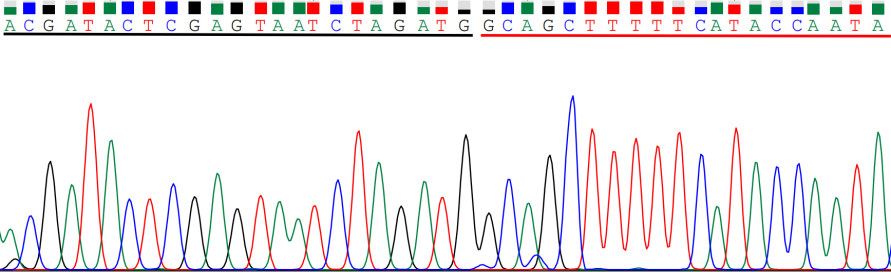


pR35BTR2-*Glyma.06g165700*

partial pR35BTR2 vector

partial *Glyma.06g165700* sequences


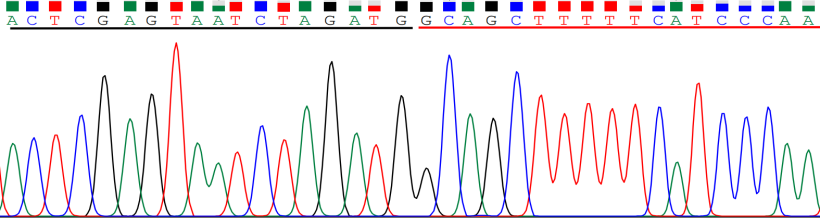


pR35BTR2-*Glyma.05g201700*

partial *Glyma.05g201700* sequences

partial pR35BTR2 vector


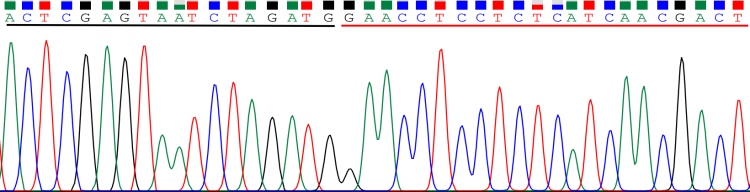


pR35BTR2-*Glyma.17g095000*

partial pR35BTR2 vector

partial *Glyma.17g095000* sequences
