# Supplementary material for: Development of a set of novel binary expression vectors for plant gene function analysis and genetic transformation
Source: Front Plant Sci. 2023 Jan 12;13:1104905. doi: 10.3389/fpls.2022.1104905 (PMC9877630; doi:10.3389/fpls.2022.1104905)
Supplement: Supplementary file 5 [file Table_1.docx]

**Table S1.** Primers used in this article

| name | Sequences (5’-3’)  (*Bsa*Ⅰ recognition sequences and sticky ends were shown in blue and yellow background, respectively. The underlined sequences are the restriction sites used in vector construction) |
| --- | --- |
| mSXb1F | GCACTCTAGATGTGAGACCGCAAGCACTCAGGGC |
| mSSa1R | GTGTCGAGCTCGGATCCTGAGACCAACCGAGCGTTCTGAAC |
| mSXb2F | GTGCTCTAGATGAGACCGCAAGCACTCAGGGC |
| mSSa2R | GACGCGAGCTCAAGCTTGCTCTGAGACCAACCGAGCGTTCTGAAC |
| mSYL1 | GTCAACTGCAGATGTGAGACCGCAAGC |
| mSYL2 | GAATTCGTTGTCAATCAATTGG |
| mSYL3 | GTCAACTGCAGATGAGACCGCAAGCACTC |
| mSHin3 | GTCACAAGCTTTGAGACCGCAAGCACTC |
| mSHi32 | GTCACAAGCTTTTGAGACCGCAAGCACTC |
| mSSa4R | GACGCGAGCTCGGATCCTAGAGACCAACCGAGCGTTCTGAAC |
| At75BF | GTGTGGTCTCTGATGGAGGGTTCGTCCAAAGG |
| At75BR | CACTGGTCTCTATCCTAATCAAATTTCACAGTCTCTCCATC |
| GUS1F | CGTAGGTCTCCTAGATGGTAGATCTGAGGGTAAATTTC |
| GUS1R | CTAGAGGTCTCAGCTCACACGTGATGGTGATGG |
| Gmb293F | GTGTGGTCTCATAGATGGGTGACAAAGAAAAGTTTGAGT |
| Gmb293R | GTCAGGTCTCAGCTCAAAGTTCAACTTTCATCTGACTTG |
| MCS41 | AGCTTCTTAAGGATCCGAATTCGGTACC |
| MCS42 | AGCTGGTACCGAATTCGGATCCTTAAGA |
| HygS1 | CATGTCGACATGAAAAAGCCTGAACTCACCGCG |
| HygS2 | CAAGTCGACTATTTCTTTGCCCTCGGACGAGT |
| KanS1 | CCTGTCGACATGGGGATTGAACAAGATGGATTGC |
| KanS2 | GAGGTCGACTCAGAAGAACTCGTCAAGAAGGCG |
| RedS1 | GAGCGTCGACATGGCCTCCTCCGAGAACGT |
| RedS2 | GAGCGTCGACCTACAGGAACAGGTGGTGG |
| GUS2F | GTCCGGTCTCTGATGGTAGATCTGAGGGTAAATTTC |
| GUS2R | GCTAGGTCTCTATCCTCACACGTGATGGTGATGG |
| 35SE1 | CGGAATTCTATTGAGACTTTTCAACAAAGGGT |
| E9tSac2 | AGCCGATTTTGAAACCGC |
| mSHin3 | GTCACAAGCTTTGAGACCGCAAGCACTC |
| mSXba1 | GTGACTCTAGAGACCAACCGAGCGTTC |
| mSHi32 | GTCACAAGCTTTTGAGACCGCAAGCACTC |
| mSXb12 | GTGATTCTAGAAAGAGACCAACCGAGCGTTC |
| AtGa4F | GTGTGGTCTCTGCTTCCACCATGGTACCATACTATTCC |
| AtGa4R | GTGTGGTCTCTATCTGGTATATATAGCTCCTGCAATTATAACA |
| GFPB1 | GTGTGGTCTCTTAGAGGTCGGATCCGGAGGTTCTATGGTGAGCAAGGGCGAG |
| GFPB2 | CTAAGGTCTCTGCTCACTTGTACAGCTCGTCCATGC |
| MSXb1F | GCACTCTAGATGTGAGACCGCAAGCACTCAGGGC |
| mSBam1 | CGAGGATCCTCCTGAGACCAACCGAGCGT |
| mSXb2F | GTGCTCTAGATGAGACCGCAAGCACTCAGGGC |
| mSBam2 | CGAGGATCCACCTGAGACCAACCGAGCGT |
| SuVvCF | GTCTGGTCTCTTAGATGGCAGCCTTTTCTCAGC |
| SuVvCR | GACTGGTCTCACACCGAAGGAGTACCTGTTAGAGAAACC |
| RfgeF | GTCTGGTCTCTTAGATGGCTTTGGGATCATGTTC |
| RfgeR | GACTGGTCTCTCACCCATCATCGTCTGATTTTCTCTTGC |
| Gm5c7F | GAGAGGTCTCTTAGATGGCAGCTTTTTCATCCC |
| Gm5c7R | GTGTGGTCTCAGCTCAATTGAAAGAACCCGAATTATT |
| Gm6c7F | GTCAGGTCTCTTAGATGGCAGCTTTTTCATACCAATAC |
| Gm6c7R | GTCAGGTCTCAGCTCGAAGTTTTGTAGGGCAGC |
| Gm2c4F | GGATGGTCTCATAGATGGATCCGGCGGCCATG |
| Gm2c4R | GTGTGGTCTCTGCTCACGTTGCTCTTTCAAAACCATC |
| Gm17c5F | GTGTGGTCTCTTAGATGGAACCTCCTCTCATCAACG |
| Gm17g5R | GTGTGGTCTCTGCTCATGTTGCTTTCTCAAAACCACC |
| mSEco1 | GCGAATTCTAGAGACCAACCGAGCG |
| mSEco2 | CGGAATTCAGAGACCAACCGAGCGTTC |
| SlEFF | TCTCCAAAGATGGTCAGACCC |
| SlEFR | CCAGAGATTGGAACAAAGGGG |
| GmActinF | GAGCTATGAATTGCCTGATGG |
| GmActinR | CGTTTCATGAATTCCAGTAGC |
| AtActinF | TCTTGATCTTGCTGGTCGTG |
| AtActinR | GAGCTGGTTTTGGCTGTCTC |
| RTGusF | TGACCATGGTAGATCTGAGG |
| RTGusR | TTTGCCTTGAAAGTCCACCG |
| At751 | TTTGTTCCATGGAGGGTTCG |
| At752 | ACCTATTCCCTAGAAGCCTATG |
